# The *Caenorhabditis elegans* Elongator Complex Regulates Neuronal α-tubulin Acetylation

**DOI:** 10.1371/journal.pgen.1000820

**Published:** 2010-01-22

**Authors:** Jachen A. Solinger, Roberta Paolinelli, Holger Klöß, Francesco Berlanda Scorza, Stefano Marchesi, Ursula Sauder, Dai Mitsushima, Fabrizio Capuani, Stephen R. Stürzenbaum, Giuseppe Cassata

**Affiliations:** 1 C. elegans Genetics, IFOM, Fondazione Istituto FIRC di Oncologia Molecolare, Milan, Italy; 2 Microscopy Center, Pharmazentrum, University of Basel, Basel, Switzerland; 3 Department of Physiology, Yokohama City University, Yokohama, Japan; 4 Computational Cell Biology, IFOM, Milan, Italy; 5 School of Biomedical and Health Sciences, Pharmaceutical Science Division, King's College London, London, United Kingdom; Stanford University, United States of America

## Abstract

Although acetylated α-tubulin is known to be a marker of stable microtubules in neurons, precise factors that regulate α-tubulin acetylation are, to date, largely unknown. Therefore, a genetic screen was employed in the nematode *Caenorhabditis elegans* that identified the Elongator complex as a possible regulator of α-tubulin acetylation. Detailed characterization of mutant animals revealed that the acetyltransferase activity of the Elongator is indeed required for correct acetylation of microtubules and for neuronal development. Moreover, the velocity of vesicles on microtubules was affected by mutations in Elongator. Elongator mutants also displayed defects in neurotransmitter levels. Furthermore, acetylation of α-tubulin was shown to act as a novel signal for the fine-tuning of microtubules dynamics by modulating α-tubulin turnover, which in turn affected neuronal shape. Given that mutations in the acetyltransferase subunit of the Elongator (Elp3) and in a scaffold subunit (Elp1) have previously been linked to human neurodegenerative diseases, namely Amyotrophic Lateral Sclerosis and Familial Dysautonomia respectively highlights the importance of this work and offers new insights to understand their etiology.

## Author Summary

We were able to demonstrate how a screen, that utilized the nematode model organism *Caenorhabditis elegans*, yielded the novel discovery that the Elongator protein complex is critical for neuronal development and microtubule acetylation. Other scientists have previously shown that the Elongator is important for transcription, but also hypothesized that a hitherto unknown function within the cytoplasm prevails. Regulation of microtubule acetylation is indeed important for cellular function. Our results provide the first tantalizing insights of how worms display neuronal phenotypes that may be linked to changes in degrees of acetylation of microtubules. We also point out that the Elongator itself has a defined acetyltransferase function that could well be directly responsible for the acetylation of α-tubulin. In conclusion, we discuss how the regulation of microtubule acetylation might impact on the understanding of human neurodegenerative diseases, namely Familial Dysautonomia and Amyotrophic Lateral Sclerosis.

## Introduction

Microtubules (MTs) are polymers of α/β-tubulin heterodimers that associate head-to-tail and laterally to form hollow tubes. MTs are usually highly dynamic and undergo rapid turnover by exchange of subunits where the ends of MTs undergo transitions between growth and shrinkage. It has been postulated that this “dynamic instability” provides a space-probing mechanism that creates contact between MTs and target organelles [1]. However, the cytoplasmic network also contains a subpopulation of stable MTs [2],[3],[4] that have an undefined cellular function, possibly required for cellular morphogenesis [5]. A distinguishing and evolutionarily-conserved feature of stable MTs is that they acquire posttranslational modifications (PTMs) in a time-dependent manner [6]. An example of a PTM is the acetylation of α-tubulin on a lysine residue at position 40. HDAC6 and SIRT2 have been shown to act as microtubule de-acetylases [7],[8],[9].

Elimination of acetylation has no obvious phenotypic consequences in *Chlamydomonas* or *Tetrahymena*, thus leading to the notion that α-tubulin acetylation is per se not required for cell survival. In *Caenorhabditis elegans* the expression of a non-acetylatable α-tubulin rescues the touch insensitivity phenotypes of neurons lacking MEC-12, the only *C. elegans* α-tubulin that contains lysine at position 40 [10],[11],[12]. However, by using other systems, acetylation of α-tubulin has been proposed to control transport mechanisms such as e.g. the dynein/dynactin transport of aggresomes [13],[14] or the selective transport of the Kinesin-1 cargo JIP1 [15]. Furthermore, MT acetylation may also be important for cell motility since overexpression of HDAC6 leads to decreased acetylation and increased cell motility, the opposite is true when HDAC6 is chemically inhibited [7],[16]. In vertebrates, a family of GTPases (Rac, Rho and Cdc42) triggers localized changes in actin and MT dynamics resulting in changes in cell motility [17]. Moreover, downstream effectors of these GTPases include the MT plus-end tracking proteins (+TIPs) that “capture” and “stabilize” the ends of MTs stimulating the detyrosination and acetylation of MTs in fibroblasts [18],[19],[20]. These data suggest that GTPases are involved in MT dynamics and PTMs. In *C. elegans* the Rac GTPase *ced-10* and the Rac-like GTPase *mig-2* have overlapping functions in axon guidance and cell migration [21], but their role in MT function has not yet been studied.

The Elongator, a component of the hyperphosphorylated holoenzyme RNA polymerase II (RNAPII), was originally identified in yeast [22]. Significantly, one subunit of Elongator, Elp3, harbors motifs found in the GNAT family of histone acetyltransferases (HATs) [23]. Both yeast and human Elongator have HAT activity in vitro, primarily directed toward histone H3 [24],[25],[26], and yeast *elp3* mutation results in decreased histone H3 acetylation levels in chromatin in vivo [24],[27]. The Elongator is associated with nascent RNA that emanates from elongating RNAPII in yeast [28] and human cells [29],[30], and thus is classified as a key component of transcript elongation. However a substantial portion of Elongator is cytoplasmic [25],[26],[31] and may function as a scaffold involved in exocytosis [32], tRNA modification [33], activation of JNK [31] or actin dynamics [34]. The precise cytoplasmic function of the Elongator remains however controversial [35]. Recently, it was reported that the Elongator can acetylate α-tubulin in vitro [36].

This work uncovers that (i) *elongator* is a regulator of α-tubulin acetylation in vivo; (ii) *elongator* is important for MT function in correct loading and velocity of vesicles in vivo and (iii) acetylation has a novel function in fine-tuning intrinsic dynamics of MTs by modulating α-tubulin turnover. Altogether, this concept adds an additional layer of understanding explaining how acetylation by the Elongator can affect MT and neuronal function.

## Results

### Hyperacetylation of MEC-12/α-tubulin in “gain of function” alleles of the rac-like GTPase *mig-2* causes neuronal phenotypes and uncoordinated movement

**Figure 1:**
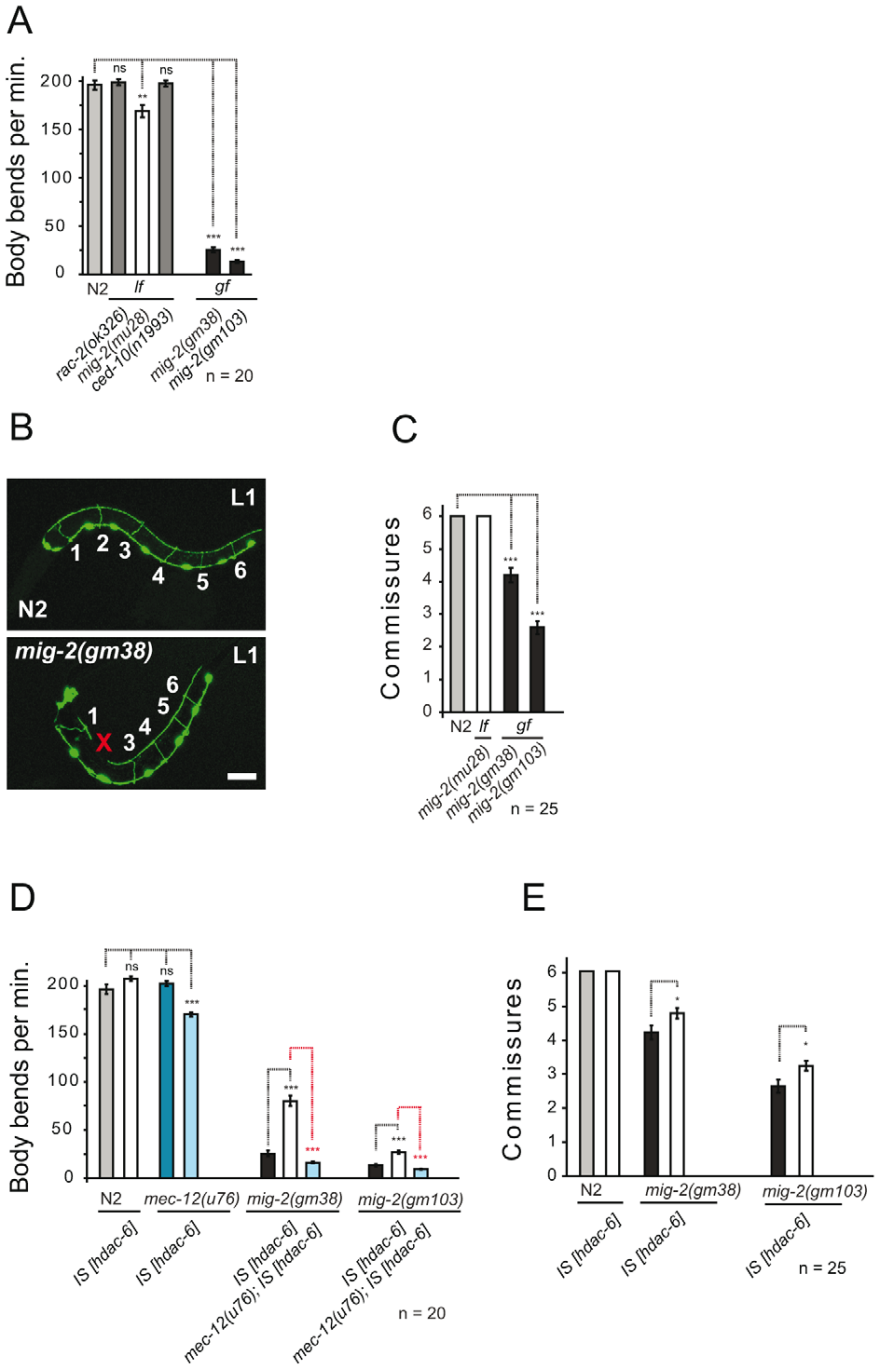
Phenotypes of *mig-2(gf)* are suppressed by overexpression of HDAC-6. (A) Movement is expressed as body bends per minute of adult wt (N2) and different alleles of different Rac GTPases. (B) Confocal micrographs depicting the missing of commissure number 2 in an *mig-2(gm38)* [*gf*] mutant L1 as compared to wt (N2) using *Punc-25::gfp* as a marker. Bar 15 µm. (C) Average commissures per L1 animal in wt (N2) and *mig-2* alleles. (D) Body bends per minute of different genetic backgrounds overexpressing HDAC-6. Synergic movements defects in *mec-12(u76)* overexpressing HDAC-6 and dependence on *mec-12* for suppression of *mig-2(gm38)* and *mig-2(gm103)* by overexpression of HDAC-6 (E) Average commissures per L1 animal overexpressing HDAC-6 in different genetic backgrounds. P values: ns = not significant; *<0,05; **<0,01; ***<0,001. Error bars: SEM.

Using various “*loss of function* (*lf*)” and “gain of function (*gf*)” alleles of the *C. elegans* Rac GTPases *rac-2*, *mig-2* and *ced-10* confirmed that *lf* and *gf* alleles of *mig-2* hamper movement as revealed by reduction in the frequency of body bends in liquid (Figure 1A, [21],[37]). VD/DD motoneurons are located ventrally and send dorsal projections called “commissures”. Using *unc-25::gfp* as a marker for these motoneurons [21], it was possible to quantify fully developed commissures in L1 larvae. Whilst wt and *mig-2(lf)* each displayed an invariable number of 6 commissures (with no differences in neuron morphology), commissures were significantly reduced in *mig-2(gf)* (Figure 1B and 1C). This supports previous reports [21],[37] that *mig-2* is required for correct body movement and that *mig-2(gf)* exerts ***unc***oordinated body movement (*unc*) and reduces commissures in VD/DD neurons.

**Figure 2:**
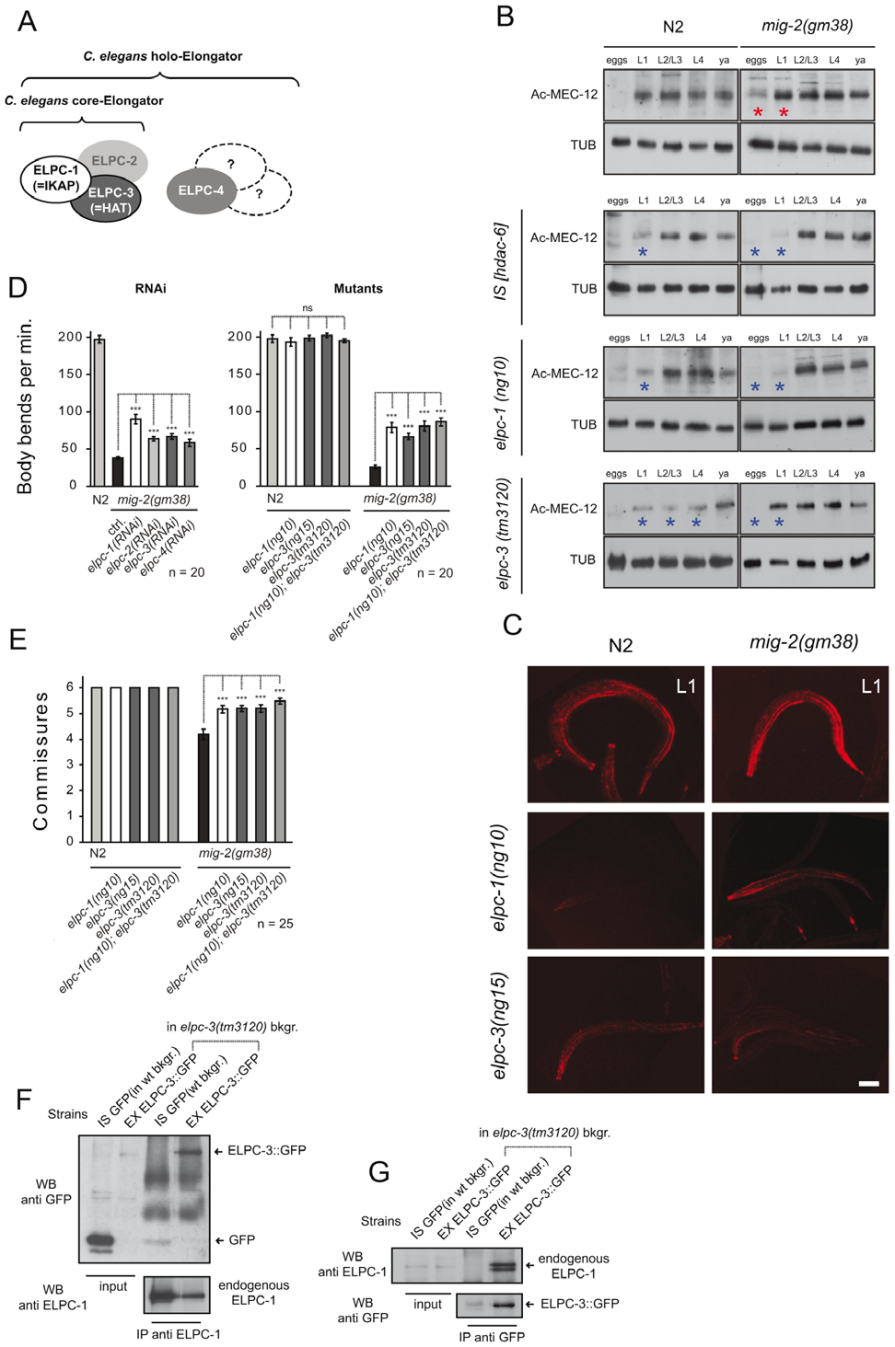
The *elongator* is required for acetylation of MEC-12/α-tubulin. (A) *C. elegans elongator* based on sequence comparison. (B) Western blot analyses of MEC-12/α-tubulin acetylation. Developmental stages of worms are indicated. Total α-tubulin (using AB directed against all α-tubulins in *C. elegans*) served as loading control. Left panels: wt (N2), HDAC-6 overexpression or *elongator* deletion mutations as indicated. Right panels: corresponding situation in the *mig-2(gf)* background. Comparative western blots of all strains to wt (N2) and *mig-2(gm38)* were carried out to calibrate the acetylation intensities, representative blots for each strain are shown. (C) Confocal micrographs of whole mount staining of acetylated MEC-12/α-tubulin in L1 larvae of different genetic backgrounds. The strongest signal is attributed to the axons of touch neurons and sensory cilia at the nose tip. Acetylation of MEC-12/α-tubulin is diminished in *elongator* mutants as compared to wt (N2) and suppresses the hyperacetylation of *mig-2(gm38)* mutants. Bar 15 µm. (D) Body bends per minute showing the suppression of *mig-2(gm38)* by *elongator* knock down by RNAi or *elongator* mutants. (E) Average commissures per L1 animal in different mutant backgrounds showing the suppression of the defects in *mig-2(gm38)* by *elongator* mutants. (F) CoIP of ELPC-3::GFP with endogenous ELPC-1 in *elpc-3(tm3120)* mutants or in GFP-expressing wt strains (*unc-25::gfp*) as a control. ELPC-1 was immunoprecipitated from protein lysates and immunoblotted with an anti-GFP antibody to detect co-precipitating ELPC-3::GFP. Inputs (1/50 of total immunoprecipitated lysate) of ELPC-3::GFP and GFP alone are shown on the left (upper panel). In the lower panel, the blot with anti-ELPC-1 antibody detects the total amount of immunoprecipitated ELPC-1. (G) Reciprocal CoIP to (F) using anti-GFP antibody for IP and anti ELPC-1 antibody for WB. P values: ns = not significant; ***<0,001. Error bars: SEM.

Rac GTPases may affect α-tubulin acetylation [19], a notion that was assessed by western blot analyses and whole mount immunofluorescence in neurons with the monoclonal antibody 6-11B-1 that specifically recognizes MEC-12, a neuronal α-tubulin that is acetylated on a single lysine residue at position 40 and that is expressed also in all motoneurons (Figure S1A, S1B, S1C, S1D, S1E, S1F, S1G). Western blot analysis uncovered that acetylation of MEC-12/α-tubulin is absent in eggs but can be reliably detected in all other stages (Figure 2B). As a control, we observed that acetylation of MEC-12 was absent in all stages within a *mec-12(e1607)* background, an allele previously described to be a putative null mutant (data not shown, [10]). Whilst wt and *mig-2(lf)* were indistinguishable from each other (data not shown), there was a marked increase of MEC-12/α-tubulin acetylation in *mig-2(gf)*, which was most pronounced in eggs and L1 stages (Figure 2B asterisks, and Figure 2C; Figure S5A for details and quantification). Although the signals were strongest in touch neurons (Figure 2C), acetylation was also shown to be present in other neurons (e.g. motoneurons, [10]). In *mec-3(e1338)*, where the touch neurons are not specified [38], the developmental acetylation pattern was analogous to wt, although slightly reduced due to the missing contribution of the touch neurons (Figure S1H and S1J). This finding confirms that acetylation of MEC-12/α-tubulin is not limited to touch neurons.

If the increase in acetylation of MEC-12/α-tubulin in *mig-2(gf)* is central to the observed body movement defects and the neuronal phenotypes, a reduction of acetylation of MEC-12/α-tubulin in a *mig-2(gf)* background should suppress the phenotypes. This was investigated by overexpressing HDAC-6, an α-tubulin deacetylase [7]. Overall acetylation of MEC-12/α-tubulin was reduced in eggs and L1 stages of wt and *mig-2(gf)* (Figure 2B). Whilst the overexpression of HDAC-6 did not cause detectable phenotypic changes in a wt background, a suppression of the *unc* phenotype (as scored by a body bends per minute assay, which simply sums the number of “left to right” and “right to left” movements of worms put into water [see also Methods], Figure 1D) and an amelioration of the neuronal morphology (Figure 1E) was apparent in the *mig-2(gf)* background (notably, the suppression of the morphological defects was not complete thus suggesting that *mig-2* may also be involved in other pathways). This finding was shown to be specific to the regulation of acetylation of MEC-12/α-tubulin, as overexpression of HDAC-6 in a *mec-12/α-tubulin* mutant background abolished the suppression of the movement defects in *mig-2(gf)*. In addition, a synthetic movement phenotype was observed in the *mec-12/α-tubulin* mutant strain overexpressing HDAC-6 (Figure 1D). Hyperacetylation of MEC-12/α-tubulin in *mig-2(gf)* can therefore be linked to the observed movement and neuronal phenotypes.

### The *elongator* is required for correct acetylation of MEC-12/α-tubulin

Based on the observation that a reduction of MEC-12/α-tubulin acetylation in *mig-2(gf)* partially (but nevertheless clearly) suppresses the movement defects, a whole genome RNAi suppression screen was performed to possibly identify regulators of α-tubulin acetylation (data not shown). We found a series of suppressors, including *elpc-1*, which upon RNAi distinctly improved the movement and neuronal phenotype of *mig-2(gf)* (Figure 2D and 2E) in an allele unspecific manner (since it suppressed the *gf* alleles: *gm38* and *gm103*, data not shown).

ELPC-1, the homologue of human IKAP/hELP1, is predicted to be a subunit of the *elongator* complex, of which (based on sequence homology) 4 members can be identified in *C. elegans*: namely *elpc-1 – 4* (Figure S2A). The core complex is made up of ELPC-1 – 3 and ELPC-4 is a member of the accessory complex (Figure 2A).

Western blot analyses of the *elongator* mutants confirmed that the overall acetylation was reduced especially in L1 stage (*elpc-1*) and L1–L4 stage (*elpc-3*) when tested in a wt background (Figure 2B blue asterisks, and Figure 2C) and in eggs and L1 in the *mig-2(gf)* background (Figure 2B blue asterisks, and Figure 2C) [for quantification of immunohistochemical data and Western blots see Figure S5]. Residual acetylation by a yet-to-be-defined pathway can be seen primarily in later stages of development.

RNAi knock-downs of individual members of the *elongator* complex demonstrated that all four were able to suppress the movement phenotypes of *mig-2(gf)* (Figure 2D). Worms harboring a chromosomal deletion of *elpc-1* and *elpc-3* or a point mutation in *elpc-3* produced by tilling (Figure S2 and Figure S3) showed no gross neuronal or movement phenotype by themselves however, all three were able to suppress the *mig-2(gf)* phenotypes (Figure 2D and 2E). Interestingly, the double mutant *elpc-1*; *elpc-3* did not enhance the suppression (Figure 2D and 2E) providing supporting evidence that *elpc-1*and *elpc-3* are not involved in parallel pathways.

A palette of translational rescue constructs (Figure S3) revealed that *elpc-1* and *elpc-3* are expressed ubiquitously. The subcellular localization of ELPC-1 is mainly in the cytoplasm, whereas ELPC-3 is predominantly nuclear but also cytoplasmatic (data not shown). This finding is analogous to the observations in human fibroblasts [25]. To test whether the core complex subunits also associate in *C. elegans*, co-immunoprecipitation assays (CoIPs) were performed with a specific polyclonal antibody directed against endogenous ELPC-1. In lysates derived from *elpc-3* deletion mutants expressing ELPC-3::GFP (Figure S4) or from wt animals expressing GFP alone, ELPC-3::GFP associated specifically with endogenous ELPC-1, while GFP alone did not (Figure 2F). These results were further confirmed by co-precipitation of endogenous ELPC-1 or ELPC-3::GFP (the reciprocal CoIPs) using antibodies against GFP (Figure 2G). We therefore conclude that the core *elongator* is conserved in worms. Besides the previously identified function in histone acetylation, this work also suggests that the *elongator* may be involved in the regulation of α-tubulin acetylation.

### The *elongator* genetically interacts with *mec-12/α-tubulin* in neurons

**Figure 3:**
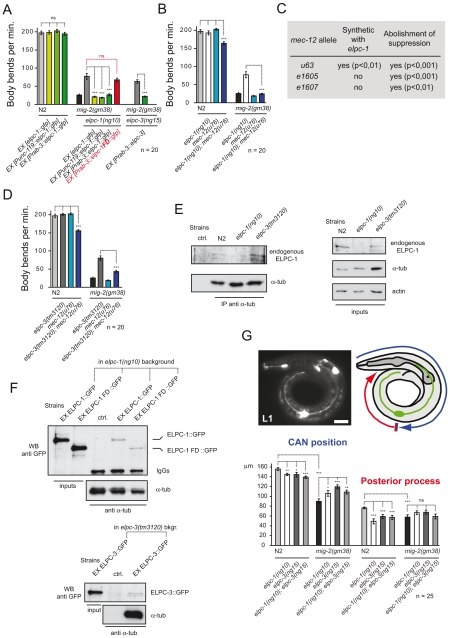
The *elongator* interacts genetically and physically with *mec-12/α-tubulin* and is required for correct neuronal morphology. (A) Rescue experiment of *elongator* mutants in different genetic backgrounds measuring body bends per minute. The *elongator* is required in neurons since the suppression of *mig-2(gm38)* is rescued by expressing *elongator* using neuron specific *unc-119* and *rab-3* promoters. Truncated Familial Dysautonomia version of ELPC-1 (*elpc-1*FD) does not rescue. (B) Body bends per minute of different genetic backgrounds showing synthetic defect of *mec-12(u76)*; *elpc-1(ng10)* double mutants and the requirement of wt *mec-12/α-tubulin* for suppression of *mig-2(gm38)* by *elpc-1(ng10)*. (C) Table of genetic interactions between *elpc-1(ng10)* and different *mec-12* alleles. Synthetic effects on movement of different *mec-12* alleles in a double mutant background with *elpc-1(ng10)*: all *mec-12* alleles abolish the suppression of *unc* phenotype in *mig-2(gm38)*; *elpc-1(ng10)* worms. (D) Body bends per minute showing synthetic defect of *mec-12(u76)*; *elpc-3(tm3120)* double mutants and the requirement of wt *mec-12/α-tubulin* for suppression of *mig-2(gm38)* by *elpc-3(tm3120)*. (E) CoIP of endogenous ELPC-1 with endogenous α-tubulin in different mutant backgrounds. α-tubulin was immunoprecipitated from protein lysates and immunoblotted with an anti-ELPC-1 antibody to detect co-precipitating ELPC-1 (left upper panel). The lower left panel shows the total amount of immunoprecipitated α-tubulin. Inputs (1/50 of total lysate) of ELPC-1, α-tubulin and actin (loading control) are shown on the upper right panels. (F) CoIP of full length or truncated (FD) ELPC-1::GFP with endogenous α-tubulin in *elpc-1(ng10)* mutants and wt strains (as a control). α-tubulin was immunoprecipitated and immunoblotted with an anti-GFP antibody to detect co-precipitating GFP tagged proteins (FD)::GFP (upper panels). Inputs (1/50 of total lysate) are shown on the left side of the panel. The blot was re-probed with anti-α-tubulin antibody to detect the total amount of immunoprecipitated protein (upper panels; IgGs: heavy chains). The lower panels show the CoIP of ELPC-3::GFP in *elpc-3(tm3120)* mutants with α-tubulin (Input: 1/50 of total lysate). (G) CAN cell position and posterior process extension measured in L1 larvae of different genetic backgrounds. Upper panels: left: fluorescence micrograph showing expression of *ceh-10::gfp* marker for the CAN neuron. Bar 10 µm; right panel: scheme explaining how measurements were performed; lower graph: average position of CAN neuron or posterior process extension. P values: ns = not significant; *<0,05; **<0,01; ***<0,001. Error bars: SEM.

Suppression of the *mig-2(gf)* phenotype by the *elongator* was analyzed in detail by re-introducing rescue constructs as transgenes into the *elongator*; *mig-2(gf)* double mutant background (constructs described in Figure S3). The translational *gfp* fusion construct of *elpc-1* was able to rescue the *elpc-1* effect in the *mig-2(gf)* background (Figure 3A). The overexpression of *elpc-1* in a wt background had no effect (Figure 3A). A similar observation was obtained with two minigene constructs, where the *elpc-1* cDNA was placed under the control of two different neuronal promoters, namely *unc-119* and *rab-3*. This denotes that the suppression of *mig-2(gf)* by *elpc-1* is due to its function in neurons. Interestingly, a truncated version of *elpc-1* (which resembles the ELP1 mutation present in patients suffering from Familial Dysautonomia (FD) [39], was not able to rescue (Figure 3A) even though its localization in the cytoplasm was unaffected by the truncation (data not shown). Finally, the P*rab-3::elpc-3* minigene in the respective *elpc-3(lf)*; *mig-2(gf)* background resulted in a complete rescue, demonstrating that *elpc-3* is required in neurons.

*elongator* mutants should behave like strains overexpressing HDAC-6, at least if acetylation is critical and therefore *elongator*; *mec-12/α-tubulin* double mutants should be equivalent to HDAC-6-overexpressing *mec-12/α-tubulin* strains. Indeed, two of the four *mec-12/α-tubulin* alleles tested (*u63* and *u76*, Table S1) showed synthetic movement defects with *elpc-1(lf)* (Figure 3B and 3C). Only the alleles with altered structure of *mec-12/α-tubulin* showed a synthetic phenotype with *elpc-1(lf)*. [This is due to the fact that *mec-12/α-tubulin* is accumulated in *elpc-1(lf)* therefore compromising neuronal function (the detailed analysis and the reasons of this synthetic phenotype are presented later in Figure 7 and Figure 8)].

Moreover, all four *mec-12/α-tubulin* alleles completely abolished the suppression in the *elpc-1(lf)*; *mig-2(gf)* double mutant (Figure 3B and 3C) thus highlighting that neurons require MEC-12/α-tubulin for *elpc-1* to correctly exert its function. The suppression of *mig-2(gf)* seems to require a precise equilibrium between MEC-12/α-tubulin and its acetylation. Interfering with this equilibrium by introducing point mutations into MEC-12/α-tubulin in fact abolishes the suppression.

Moreover, a genetic interaction was observed in the *elpc-3(lf)*; *mec-12/α-tubulin* double mutant. Furthermore, the suppression of *mig-2(gf)* by *elpc-3* required wt *mec-12/α-tubulin* (Figure 3D). Taken together, these findings reveal that the *elongator* is likely a regulator of neuronal function mediated by MEC-12/α-tubulin.

The movement phenotypes analyzed here are independent from touch neuron function where *mec-12/α-tubulin* is required [10]. This is confirmed by the fact that *elpc-1* can suppress *mig-2(gf)* phenotypes in a *mec-3(e1338)* background, where the touch neurons are not specified (Figure S1I). Moreover, *elpc-1(lf)* and *elpc-3(lf)* are normal in touch sensitivity (Figure S4A) and it has been shown that acetylation of *mec-12/α-tubulin* is not critical for touch sensitivity [10].

Besides neuronal function, *mig-2* is also involved in vulva development and *mig-2(gf)* alleles are ***eg***g ***l***aying defective (*egl*) [37]. In isolation, *elongator* alleles did not display notable egg laying defects and the *elongator*; *mig-2(gf)* strains could not suppress the *egl* phenotype (data not shown). Furthermore, it is known that *mig-2* is required for correct distal tip migration and phagocytosis of apoptotic cell corpses in the gonad [21],[40]. Various combinations of *elongator*, *mig-2* and *mec-12/α-tubulin* mutants were tested, but no phenotypes were observed (Table S2 and Table S3). This suggests that *elongator* and *mec-12/α-tubulin* are not required for vulval development, distal tip migration or cell corpse phagocytosis.

### Molecular association of the *elongator* to α-tubulin

Endogenous ELPC-1 and α-tubulin co-immunoprecipitated in a wt and *elpc-3* deleted background (Figure 3E). This suggests that the ELPC-1/α-tubulin association is independent of ELPC-3. The association between ELPC-1 and α-tubulin was further analyzed by expressing full length and FD truncated GFP tagged versions of ELPC-1 in *elpc-1(lf)* mutants followed by the CoIP of α-tubulin. A similar result with α-tubulin was observed with both variants of ELPC-1::GFP (Figure 3F). This is not surprising since FD-ELPC-1::GFP localized like the full length protein. ELPC-3::GFP was also shown to co-immunoprecipitated with α-tubulin (Figure 3F), while GFP alone did not (data not shown). Immunoprecipitation with 6-11B-1, the antibody directed against acetylated α-tubulin, revealed that ELPC-1::GFP did not associate with the acetylated form of α-tubulin (data not shown). This suggests that the affinity of ELPC-1 to α-tubulin is lost after acetylation.

The observed genetic interaction between *elongator* and *mec-12/α-tubulin*, the physical association of the core Elongator and α-tubulin and the fact that MEC-12/α-tubulin is less acetylated in *elongator* mutants suggest that *elongator* plays a role in α-tubulin acetylation.

### Reduction of acetylation of Lysine 40 in neuronal *mec-12/α-tubulin* is critical for the suppression of *mig-2(gf)*

**Figure 4:**
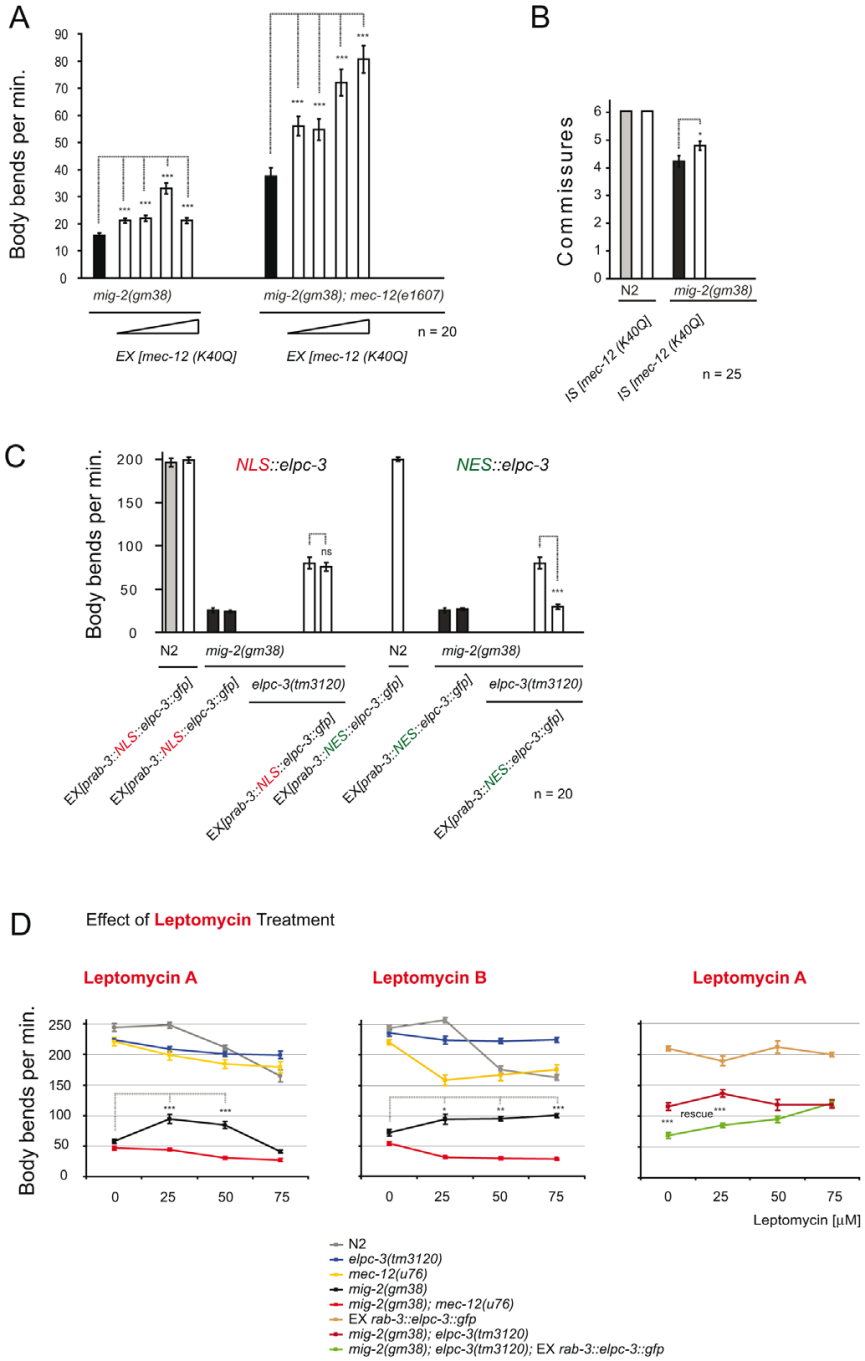
Effects of *mec-12*/*α-tubulin*[K40Q] and cytoplasmic requirement of ELPC-3. (A) *mig-2(gm38)* or *mig-2gm38);mec-12(e1607)* animals were injected with 3ng/µl of the *gfp::mec-12[K40]* construct. The transgene suppresses the movement phenotypes of both genetic backgrounds. 4 different lines with increasing *gfp* signal were tested. (B) Expression *gfp::mec-12[K40]* suppresses the defects in commissures of *mig-2(gm38)*. (C) Expression of *NES::elpc-3* (nuclear export signal) but not *NLS::elpc-3* (nuclear localization signal) rescues the suppression in *mig-2(gm38);elpc-3(tm3120)* double mutants. (D) Leptomycin treatment of worms with different genetic backgrounds. Leptomycin A and B suppress the movement defects of *mig-2(gm38)*. The suppression is dependent on *mec-12*, since *mig-2(gm38);mec-12(u76)* cannot be suppressed. Rescue of the suppression in the *mig-2(gm38)*; *elpc-3(tm3120)* EX [*elpc-3::gfp*], is abolished at high concentrations of Leptomycin A. P values: ns = not significant; *<0,05; **<0,01; ***<0,001. Error bars: SEM.

To further analyze the role of acetylation of *mec-12/α-tubulin* in neurons of *mig-2(gf)* animals we expressed *gfp::mec-12/α-tubulin* mutated at lysine 40 (K40Q). Overexpressing a non acetylable *gfp::mec-12/α-tubulin* in *mig-2(gf)* should out-compete the overacetylated endogenous *mec-12/α-tubulin* and therefore reduce the overall acetylation. This should suppress the *mig-2(gf)* phenotypes. The results shown in Figure 4A show how expression of the *gfp::mec-12/α-tubulin K40Q* transgene suppresses the *mig-2(gf)* phenotypes in a dose dependent manner. High doses are deleterious, as was also seen with an integrated version of *mec-12/α-tubulin K40Q* used in Figure 4B and Figure 7A and 7C (data not shown). *elongator* mutants should behave like strains overexpressing HDAC-6, at least if acetylation is critical and therefore *elongator*; *mec-12/α-tubulin* double mutants should be equivalent to HDAC-6-overexpressing *mec-12/α-tubulin* strains. Indeed, two of the four *mec-12/α-tubulin* alleles tested (*u63* and *u76*, Table S1) showed synthetic movement defects with *elpc-1(lf)* (Figure 3B and 3C). Only the alleles with altered structure of *mec-12/α-tubulin* showed a synthetic phenotype with *elpc-1(lf)*. [This is due to the fact that *mec-12/α-tubulin* is accumulated in *elpc-1(lf)* therefore compromising neuronal function (the detailed analysis and the reasons of this synthetic phenotype are presented later in Figure 7 and Figure 8)].

The dose of *gfp::mec-12/α-tubulin K40Q* required for suppression of *mig-2(gf)* in a *mig-2(gf);mec-12(e1607)* double background appears to be higher than in a single *mig-2(gf)* mutant. We hypothesize that the difference observed in the two backgrounds is due to negative effects of MEC-12/α-tubulin overexpression. In the *mig-2(gm38)* background, the total levels of functional MEC-12/α-tubulin protein are higher, because of the concomitant presence of the endogenous *mec-12* gene. For this reason the *mig-2(gm38)* worms reach a threshold of toxicity at lower *mec-12/a-tubulin* array expression levels. The general reduction of movement seen in the left panel in *mig-2(gm38)* is probably due to negative effects of the array in this background. Moreover, the expression of *gfp::mec-12/α-tubulin K40Q* also suppressed the defects in commissure formation (Figure 4B). This indicates a possible role of *mec-12/α-tubulin* acetylation during neuronal development. We conclude that acetylation on MEC-12 K40 is an important (but probably not the sole) aspect of the *elongator* phenotype.

### Cytoplasmic requirement of the ELPC-3

To study the cytoplasmic function of the Elongator, neuronally expressed *elpc-3* were tagged with the well characterized nuclear localization signal [NLS] (to localize *elpc-3* into the nucleus) or with a nuclear export signal [NES] (in order to direct the expression mainly in the cytoplasm). No well-established NES has been characterized in worms yet. NESs are leucine-rich sequences with an evolutionary conserved consensus LX_1–3_ LX_2–3_ LXL [41].

For the purpose of this study the well known NES of HIV Rev protein was chosen. If *elpc-3* is required in the cytoplasm to exert its function with *mec-12/α-tubulin* then only the NES version, but not the NLS version, should be able to rescue. The correct nuclear and/or cytoplasmic localization was assayed by microscopy since both construct are translational *gfp* fusions and expressed in predicted subcellular compartment (data not shown). Figure 4C shows the body bends assays confirming the hypothesis that NES::ELP-3 was able to rescue the suppression in the *mig-2(gf)*; *elp-3(lf)* background, whereas NLS::ELP-3 was not.

To further confirm the cytoplasmic requirement of the Elongator in our system we carried out body bends assays of worms treated with Leptomycin A and B. Leptomycin B has previously been shown to be an important tool in the study of nuclear-cytoplamic transport in *C. elegans* [42]. The molecular composition of Leptomycin A is very similar to Leptomycin B and both were used to substantiate the results. We hypothesized that treating *mig-2(gf)* animals with Leptomycin would sequester the Elongator to the nucleus and therefore reduce the acetylation of *mec-12/α-tubulin*, which in turn should suppress the body bends defects of *mig-2(gf)*. Worms were raised in the presence of the drugs, so ensuring that possible developmental effects were not excluded when the locomotion tests were performed (at the young adult stage). The correct nuclear localization of *elpc-3* upon drug treatment was assayed by microscopy using a transgenic strain bearing a translational rescuing *elpc-3::gfp* fusion which correctly localized to the nucleus in worms treated with Leptomycin (data not shown). The movement data obtained not only confirmed the hypothesis (Figure 4D), but also show that the suppression of *mig-2(gf)* is dependent on *mec-12/α-tubulin*, since the suppression was abolished in a *mig-2(gf)*; *mec-12/α-tubulin* double mutant.

Rescue experiments were performed using neuronally expressed *elpc-3::gfp* in *elpc-3(lf)*; *mig-2(gf)* double mutants. As seen in Figure 4D, the *elpc-3::gfp* construct is functional. Furthermore, this experiment confirmed the cytoplasmic requirement of the Elongator, since the rescue was progressively lost by increasing the concentration of Leptomycin A (Figure 4D). The effect of Leptomycin A was persistently higher than Leptomycin B, which was toxic at high concentrations (data not shown).

### The *elongator* is required in CAN neurons for proper cell migration and posterior process outgrowth

It has been shown that *mig-2* is important for cell migration and axon pathfinding in the CAN neuron (Lundquist et al and references therein [21]). Since the body sizes of *elongator* mutants and wt worms are identical, the position of the CAN cell body was measured to assay cell migration and posterior process outgrowth in L1 larvae (Figure 3G). In *elpc-1* and *elpc-3* mutants the CAN cell migrated less and the posterior process was shorter than in wt. In the *elpc-1*; *elpc-3* double mutant the phenotypes were not enhanced, indicating that *elpc-1* and *elpc-3* are not involved in parallel pathways (Figure 3G). No misguidance or branching phenotypes were observed. Notably, *elongator* mutants were able to ameliorate the CAN cell migration defect, but not the posterior process outgrowth of *mig-2(gf)* (Figure 3G). These results suggest that the *elongator* is required for correct neuronal migration and axonal extension.

### The velocity of Dense Core Vesicles is regulated by *elongator*

**Figure 5:**
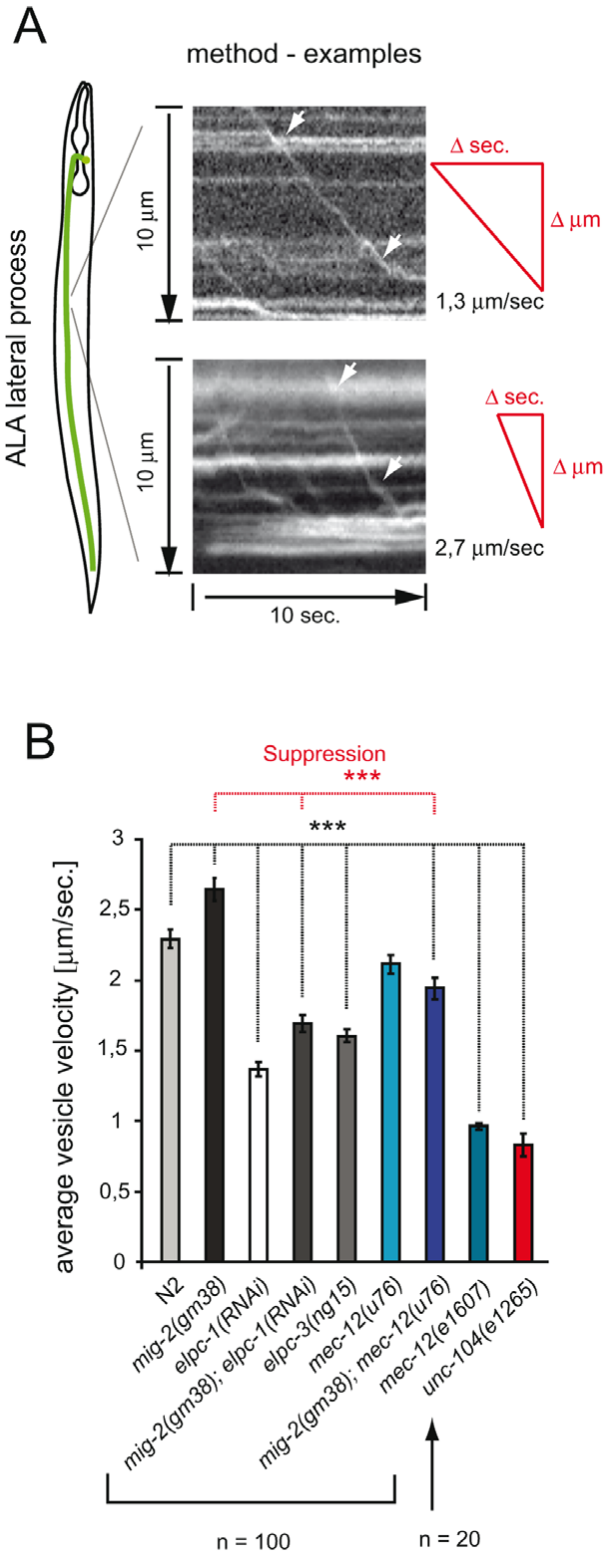
Velocity of Dense Core Vesicles is regulated by acetylation of α-tubulin. (A) Scheme depicting how kymographs of vesicles marked by IDA-1::GFP were obtained from ALA neurons of adult animals to measure the velocity. One slow vesicle (1,3 µm/sec) and one faster vesicle (2,7 µm/sec) are shown as examples. (B) Graph summarizing mean velocities in different genetic backgrounds. Mean velocities reflect the degree of acetylation of α-tubulin. *mig-2(gm38)* is faster, *elongator* mutants/RNAi are slower. *mec-12(u76)* can modulate the *mig-2(gm38)* velocity phenotype. Likewise *elpc-1(RNAi)*, *mec-12(e1607)* and *unc-104(e1265)* [example for slow vesicles [43]]. P values: ***<0,001. Error bars: SEM.

Acetylation of MTs has been proposed to be important for vesicle transport [15]. In *C. elegans* a unique tool to measure vesicle velocities exists: namely IDA-1::GFP which marks the dense core vesicles in some neurons [43]. The velocity of dense core vesicles (DCVs) along MT tracks was measured in the ALA lateral process of living worms using a fast time-lapse method [43] (Figure 5A). If the acetylation of MT positively regulates DCV velocity, then a reduced function of the *elongator* should slow down the vesicles. The mean velocity of vesicles measured in wt animals reflected the published data for wt animals (2,25 µm/sec. vs 2,10 µm/sec. [43]). Indeed, reducing the function of the *elongator* diminished the average velocity of DCVs by about 35% of wt young adults (Figure 5B). In contrast, the mean velocity of *mig-2(gf)* mutant was increased by about 13%. In addition, the phenotype of *mig-2(gf)* was suppressed in the *mig-2(gf)*; *elpc-1(RNAi)* double mutant. Analogous to this, the increased velocity of *mig-2(gf)* was suppressed by *mec-12(u76)*, even though *mec-12(u76)* on its own had no velocity phenotype (Figure 5B). Moreover *mec-12(e1607)* in which mec-12 is not acetylated showed a marked reduction of DCV velocity (Figure 5B). These experiments confirm that a pathway with *mig-2*, *elongator* and *mec-12* affects vesicle transport in vivo. It is known that transport of DCV relies, in part, on the same mechanisms as the transport of clear core vesicles [43]. Clear core vesicles transport neurotransmitters are important for movement (see below), however, tools to measure the transport of clear core vesicles directly are not available yet. Since MT are also acetylated in motoneurons, acetylation may play a role in regulating transport of clear core vesicles and therefore also movement.

### The acetylcholine concentration is decreased at synaptic clefts of neuromuscular junctions in *elongator* mutants

**Figure 6:**
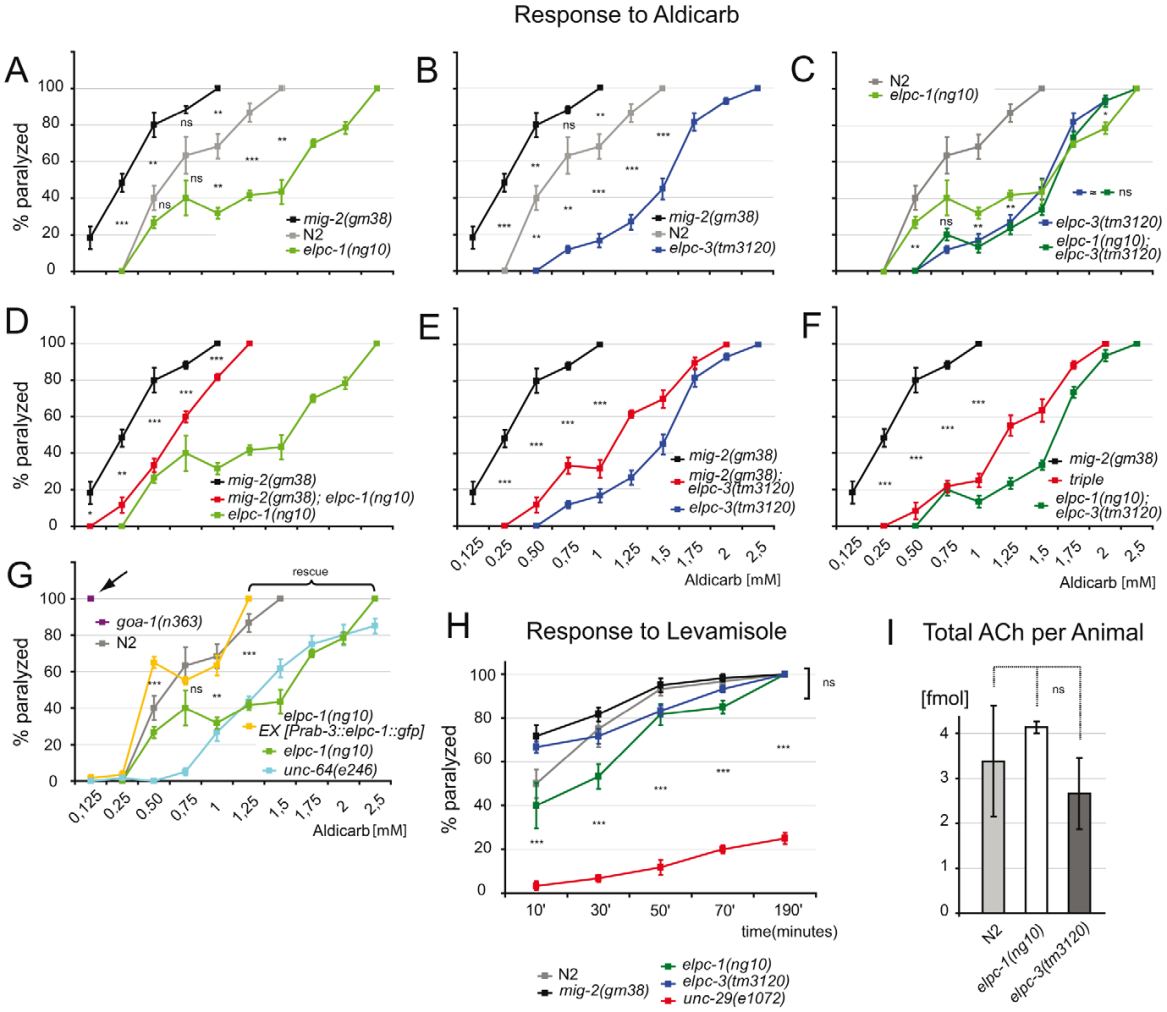
Response to aldicarb and levamisole. (A) Percentage of paralyzed adult animals in response to aldicarb. Each point of the curve represents the average of 5 experiments. *mig-2(gm8)* are more sensitive than wt(N2) and *elpc-1(ng10)* is resistant. p value is calculated for each drug concentration. (B) *elpc-3(tm3120)* are aldicarb resistant. (C) *elongator* double mutants are not significantly different from single mutants. (D) *elpc-1(ng10)* suppresses *mig-2(gm38)*. (E) *elpc-3(tm3120)* suppresses *mig-2(gm38)*. (F) *elpc-1(ng10)*; *elpc-3(tm3120)* suppresses *mig-2(gm38)* in a triple mutant. (G) Neuronal expression of *elpc-1* rescues the aldicarb resistance of *elpc-1(ng10)*. The resistance to aldicarb of *elpc-1(ng10)* under these conditions is similar to syntaxin mutants (*unc-64(e246)* [44]). Classical mutants that are hypersensitive to aldicarb are severely more sensitive than *mig-2(gm38)*. Arrow points to the already fully paralyzed phenotype at low concentration of the *hic* mutant *goa-1(n363)* [45]. (H) Response to 100 µm levamisole to test the postsynaptic apparatus. All genotypes tested are similar to wt(N2). (I) Amount of acetylcholine per adult animal determined by chromatography. P values: ns = not significant; *<0,05; **<0,01; ***<0,001. Error bars: SEM.

In *C. elegans* movement is controlled by the major neurotransmitter acetylcholine (ACh). Aldicarb, a potent ACh esterase inhibitor that potentiates ACh response, can be used to test for changes in ACh concentration at neuromuscular junctions (NMJs). In a standard test, which utilizes different concentrations of aldicarb, the sensitivity of adult *elongator* mutants and wt were identical ([44]; and data not shown). However, a differential sensitivity towards aldicarb was observed when exposure time was reduced from 4 hours to 1 hour. Compared to wt *elpc-1*, *elpc-3* and the *elongator* double mutant less sensitive to aldicarb (Figure 6A–6C). The double mutant was indistinguishable from single mutants (Figure 6C). All *elongator* mutants suppressed the aldicarb phenotype of *mig-2(gf)* mutants (Figure 6D–6F). The sensitivity of *mig-2(gf)* was not as pronounced as in *goa-1(n363)* [a classical hypersensitive mutant [45] that displays a strong phenotype (Figure 6G)], this might at least in part be due to developmental defects. The resistance to aldicarb of *elpc-1* mutants was comparable to the syntaxin mutant *unc-64(e264)* (Figure 6G). *elpc-1(lf)* could be rescued by re-expressing *elpc-1* in neurons (Figure 6G). This proves that pre-synaptic *elpc-1* is required to ensure correct ACh concentrations at NMJs. The reason for the reduction of ACh at NMJs might in part be due to reduction of velocity of vesicles. On the other hand the hypersensitive phenotype of *mig-2(gm38)* might be due to the overacetylation. Although this is a possible explanation it has to be considered that developmental defects in *mig-2(gm38)* might further influence the phenotype (e.g. by wrong NMJ wiring). Since no method exists to date to directly measure the ACh flow towards the NMJs other functions than acetylation may be additionally responsible for the *mig-2(gf)* phenotype in these tests.

Using levamisole, an agonist of the nicotinic ACh receptor [44], it was possible to assess neurotransmission and function of the postsynaptic apparatus in *elongator* mutants. The response of adult *elongator* mutants and *mig-2(gf)* animals was statistically equal to wt but clearly different to *unc-29(e1072)*, a mutant with a defective nicotinic ACh receptor (Figure 6H). These data underline the notion that the *elongator* and the *mig-2(gf)* mutants regulate ACh at NMJs due to presynaptic defects. Interestingly the degree of sensitivity to aldicarb correlated with the level of acetylation of the MTs. Whether changes of ACh levels at NMJs is a result of the direct or indirect regulation of acetylation of microtubules and changes of vesicle behavior remains to be analyzed in other systems. To verify that the change of ACh at NMJs is not due to a defect in ACh synthesis, a chromatographic method was devised capable of measuring ACh concentrations directly in worm extracts. The amount of ACh per adult worm was determined to approximate 3 fmol, a concentration statistically invariable in the *elongator* mutants (Figure 6I). Therefore the phenotypes are unlikely a result of differences in presynaptic ACh production.

### Acetylation of Lysine 40 fine-tunes the levels of MEC-12/α-tubulin and resistance of microtubules to nocodazole

**Figure 7:**
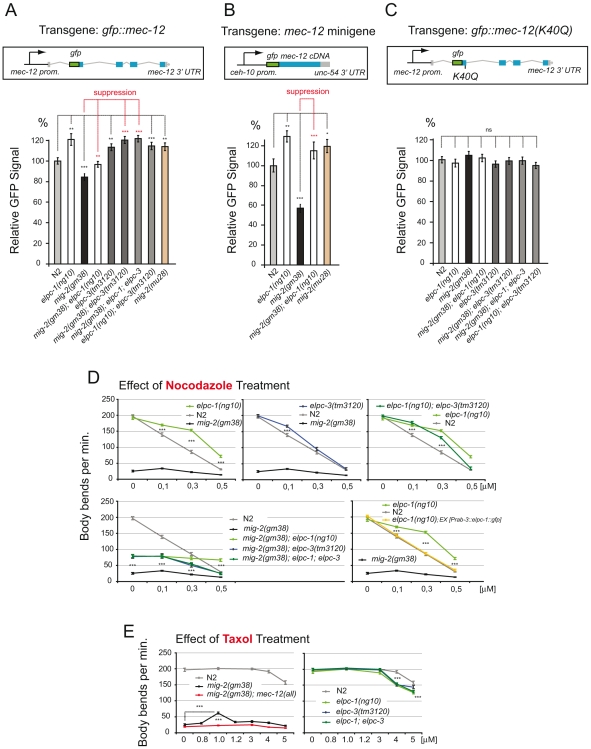
Regulation of MEC-12/α-tubulin levels depends on acetylation; response to nocodazole and taxol. (A) Regulation of GFP::MEC-12 relative signal depending on the genetic background. Genetic interactions are depicted. Construct: Integrated translational *gfp* fusion; n>35. GFP signals measured in ALM neuron cell body. (B) Regulation of relative GFP::MEC-12 signal is conserved when expression is driven from an integrated *gfp::mec-12* cDNA minigene containing a heterologous promoter with a heterologous 3′ UTR; n = 25. GFP signals measured in CAN neuron cell body (C) Abolishment of regulation of GFP::MEC-12 relative signal when lysine 40 is mutated. Construct: Integrated translational *gfp* fusion with point mutation (K40Q); n>35. GFP signals measured in ALM neuron cell body. (D) Effect of nocodazole treatment. Body bends per minute were counted at the indicated concentrations. Upper panels show resistance of *elongator* mutants. Lower left: effects of *elongator* mutants in the *mig-2(gf)* background. Lower right: rescue of nocodazole resistance by neuron specific *elpc-1* expression (n = 20). (E) Effect of taxol treatment. Body bends per minute were counted at the indicated concentrations. Left panel: suppression of *mig-2(gf)* movement defects by specific taxol concentration. Right panel: taxol sensitivity of *elongator* mutants (n = 20). P values: ns = not significant; *<0,05; **<0,01; ***<0,001. Error bars: SEM.

Even though elongator and *mec-12/a-tubulin* were shown to interact genetically (Figure 3), DNA microarray and RT-PCR of *elpc-1(ng10)* and wt revealed no differential regulation of *mec-12/α-tubulin* transcripts (data not shown). The levels of MEC-12/α-tubulin were analysed via a functional GFP-tagged version (to date a specific antibody to endogenous MEC-12 has not been raised). Surprisingly, the amount of GFP::MEC-12 protein was found to be inversely proportional to the degree of acetylation (the cell body of the touch neuron ALM was chosen to measure the GFP signal in L1 of all genetic backgrounds). In detail, compared to wt, the GFP signal of *elongator* and *mig-2(gf)* mutants was about 20% higher and 20% lower, respectively (Figure 7A). The reduced level of GFP::MEC-12 signal observed in *mig-2(gf)* mutants could be suppressed by crossing in *elongator* mutants (Figure 7A). The double mutant *elpc-1*; *elpc-3* did not enhance the effect (Figure 6A), suggesting that *elpc-1* and *elpc-3* act together in this function. We wanted to investigate whether this effect is transcriptional or posttranslational. The first evidence that this regulation is posttranslational was provided by the fact that GFP::MEC-12 cDNA driven by a heterologous promoter with a heterologous 3′ UTR did not change the relative levels of GFP::MEC-12. This analysis was performed using the CAN-neuron specific promoter *ceh-10* (to rule out possible effects of differences in *mec-12* expression in mechanosensory and non-mechanosensory neurons). As before, the signal from this GFP::MEC-12 minigene was increased in the *elpc-1* and *mig-2(gf)*; *elpc-1* background, but diminished in the *mig-2(gf)* background (Figure 7B). In a previous experiment, a point mutation that changed lysine 40 to glutamine (Q) in MEC-12/α-tubulin was still able to rescue a touch sensitivity phenotype, therefore MEC-12(K40Q) was considered to be structurally functional [10]. Follow up experiments presented here were used to investigate whether lysine 40 has an impact on the regulation of GFP::MEC-12 levels. Given that the regulation was abolished in GFP::MEC-12(K40Q) transgenic animals (Figure 7C), provides strong evidence that the acetylation of lysine 40 is important for the posttranslational fine-tuning of MEC-12/α-tubulin levels presented here.

**Figure 8:**
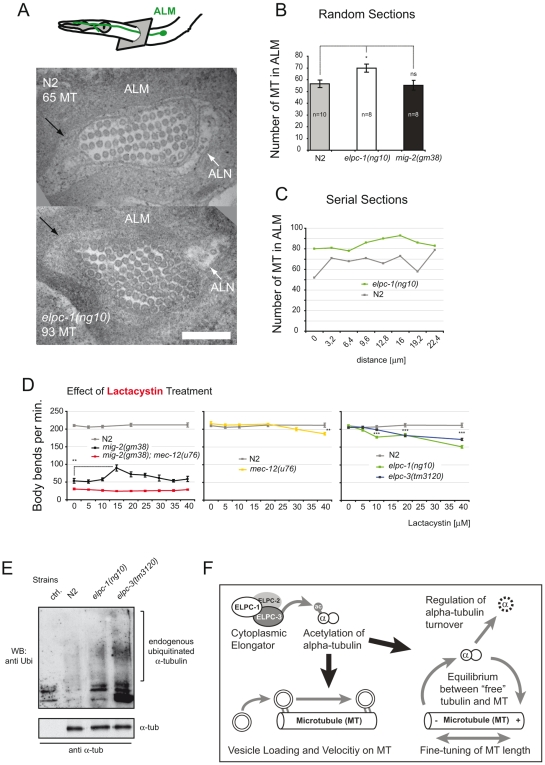
Microtubule structure and proteasome inhibition; model. (A) TEM micrograph depicting cross-sections of wt and *elpc-1(ng10)* ALM anterior neuronal process. ALN neuron is indicated. Black arrow points to the “ALM mantle”. Genotype and number of MT cross-sections are indicated. Bar 200 nm. (B) Number of MT cross-sections in ALM in random sections of different animals. (C) Number of MTs in serial sections of one animal to determine qualitatively variation in number along the axon. (D) Body bends per minute in response to lactacystin treatment. N2 movement is unaffected. Lactacystin can suppress the *mig-2(gm38)* phenotype. Suppression is dependent on *mec-12/α-tubulin*, since it is abolished in *mec-12(u76)*; *mig-2(gm38)* background (first panel). *mec-12* and *elongator* mutants are susceptible to high concentrations of lactacystin (second and third panel). (E) Endogenous α-tubulin from different mutant backgrounds was immunoprecipitated with anti- α-tubulin antibody and immunoblotted with an anti-Ubi antibody to detect modification of the protein by endogenous Ubi (upper panel). Lower panel: total amount of immunoprecipitated α-tubulin. (F) Model. P values: ns = not significant; *<0,05; **<0,01; ***<0,001. Error bars: SEM.

These observations also explain the unexpectedly high levels of acetylation in L4 and young adult worms. This might be due to the elevated amount of MEC-12/α-tubulin levels leading to a misinterpretation of the Western data. In fact we observe an accumulation of MEC-12/α-tubulin in adults (see Figure 8).

To decipher whether the increase of MEC-12/α-tubulin alters dynamics of MTs in vivo, the movement phenotype was scored in worms grown in the presence of sublethal concentrations of the MT depolymerizing drug nocodazole or the MT stabilizing drug taxol [46]. This assay was designed to uncover possible effects of the drug during development, e.g. commissure formation defects that persist from L1 to adulthood (since the worms developed through all stages in the presence of the drug). In wt, movement was impaired by nocodazole in a linear dose dependent manner. *elpc-1* and to a lesser extent *elpc-3* mutants were both more resistant to nocodazole treatment with no additive effect seen in the double mutant *elpc-1*; *elpc-3* (Figure 7D). Compared to *mig-2(gf)*, the suppression of *mig-2(gf)* by single or double *elongator* mutants resulted in an increased resistance to nocodazole (Figure 7D). To gain an insight into whether the observed nocodazole resistance in *elongator* mutants is neuron specific or due to a broader effect, *elpc-1* was re-introduced under the control of a neuron specific promoter into *elpc-1* mutant worms. The resistance to nocodazole was abolished and essentially wt, suggesting that this phenotype of *elpc-1* is neuron specific (Figure 7D).

If stabilization of MTs is fundamental for the suppression of *mig-2(gf)* by *elongator* mutants (as suggested by the nocodazole experiment) then MT stabilization by taxol should have a similar effect. In turn, *elongator* mutants may be expected to have an increased sensitivity towards taxol. Indeed, exposure to taxol suppressed the movement phenotype in *mig-2(gf)*, an effect that was dependent on *mec-12/α-tubulin*. In contrast, *elongator* mutants by themselves displayed an increased sensitivity to high concentrations of taxol (Figure 7E). In conclusion, these experiments show that *elongator* fine-tunes the amount of MEC-12/α-tubulin protein, which in turn may regulate the sensitivity of MTs to depolymerizing and stabilizating drugs revealing a change in their dynamic properties.

### Regulation of microtubule structure in *elongator* mutants

Resistance to nocodazole in *elpc-1(lf)* may arise due to MTs being stabilized by MT associated proteins. However this is unlikely, as the comparative expression analysis of wt and *elpc-1* mutants by DNA microarray technology yielded no MT associated proteins (data not shown). Alternatively, MTs number or length may be increased, a notion that was addressed by transmission electron microscopy (TEM). An ideal target for investigation proved to be the ALM neuron because firstly, it has an unusually high number of special MTs (Figure 8A) and secondly, MEC-12/α-tubulin is highly expressed [10],[47]). In randomly selected TEM sections, the overall number of MTs was indistinguishable in wt and *mig-2(gf)* but significantly increased in *elpc-1(lf)* (Figure 8A and 8B). This was confirmed in serial sections (in 3.2 µm increments) of single wt and *elpc-1(lf)* mutant worms (Figure 8C). The identified variation in MT number along the axon was fed into a mathematical model (see Experimental Procedures for details) which suggested that, the difference identified in *elpc-1(lf)* may be also the result of MTs being longer rather than solely based on an increase of MT number (Figure S5B). This is, as said above, further supported by the fact that the DNA microarray data could not pinpoint MT nucleation factors (e.g. γ-tubulin) in *elpc-1(lf)* that are instrumental for the increase in MT numbers. In summary, this provides at least circumstantial evidence that an increase in MTs number is unlikely (Figure 8A–8C).

If increasing the amount of MEC-12/α-tubulin causes longer MTs and subsequently suppresses *mig-2(gf)* movement defects, any alternative means that increases MEC-12/α-tubulin levels should be able to replicate these observations. Treatment with lactacystin, a potent and specific inhibitor of the proteasome used in *C. elegans* [48], imposed no movement effect on wt animals, but was able to partially suppress the movement phenotype of *mig-2(gf)* at 15 µM (Figure 8D). As mentioned before, the treatment was continuous during development of the worms, thereby ensuring that defects during early larval stages impose effects on locomotion at young adult stage. Moreover, the suppression was dependent on MEC-12/α-tubulin (Figure 8D), thus (re)confirming the taxol data (Figure 7E). Interestingly, compared to wt, *mec-12/α-tubulin* and *elongator* mutants were all sensitive to high concentrations of lactacystin (Figure 8D). In both cases high levels of lactacystin are predicted to impair MT function, where accumulation of a mutated MEC-12/α-tubulin and “overstabilized” MTs in *elongator* mutants alter MT function; findings that are analogous to the taxol experiments (Figure 7E).

Ubiquitin mediated proteolysis is the key player in the control of cytoplasmic protein turnover. To investigate whether impaired proteasomal proteolysis of α-tubulin might be linked to the phenotypes observed in *elongator* mutants, immunoprecipitated α-tubulin from different genetic backgrounds was probed with an anti ubiquitin antibody (Figure 8E). The ubiquitination level was higher in *elpc-3* mutants compared to worms with an *elpc-1* background. Interestingly, this observation parallels the strong effects on acetylation levels in *elpc-3* mutants (Figure 2B). These differences in post-translational modification are possibly due to the fact that ELPC-3 is the catalytic subunit of the elongator complex. This experiment demonstrated that α-tubulin is strongly modified by endogenous ubiquitin in *elongator* mutants. We conclude that *elongator* is a critical factor for α-tubulin turnover.

## Discussion

### *elongator* regulates α-tubulin acetylation

The results presented here demonstrate how a genetic animal model can be used to study acetylation of MTs. In detail, it was possible to corroborate that *mig-2* regulates α-tubulin acetylation and ascertain that the *elongator* is a new regulator of α-tubulin acetylation. Recently, Creppe and colleagues showed that elongator is involved in this process in mouse cortical neurons [36]. Their data confirm the hypothesis, presented here, namely that elongator activity regulates α-tubulin function. In our work, regulation of acetylation by the *elongator* was shown to be strong in early stages of development and acetylation of α-tubulin lower, but nevertheless still present, in late stages of *elongator* mutants. The fact that *elongator* also regulates other processes and that some acetylation remains in the absence of *elongator* suggests the presence of some degree of redundancy. It should be noted that during embryonic development and early larval stages numerous neurons undergo dramatic morphological change and in consequence, a tight control of acetylation by *elongator* has a deep impact on neuronal structure during development. This notion is discussed below.

### Regulation of MT acetylation and neuronal shape

Gain of function alleles of the Rac-like GTPase *mig-2* display defects in neuronal shape due to the hyperacetylation of α-tubulin. The defects are partially suppressed (although not fully) when acetylation is reduced either by overexpression of HDAC-6, the α-tubulin deacetylase, or by knocking out the *elongator*. This implies that *mig-2* modulates other pathways as well. For example *mig-2* is also involved in actin dynamics to regulate neuronal shape and axon pathfinding [49]. But if acetylation of MTs is regarded as a distinct fact, how can acetylation of MTs affect cell shape? It has been shown in fibroblasts and neurons that Rac GTPases regulate MT dynamics by different mechanisms. One of those is the recruitment of +TIPs such as APC and EB1 to promote stabilization via Rac effector mDia [17]. The same +TIPs are proposed to act in the linkers between actin cortex and the protruding MTs in the growth cone of neurons [50]. Although acetylation of MTs seems to be secondary to stabilization [6], it has not been excluded that RacGTPases may actively regulate acetylation.

If α-tubulin is hyperacetylated, as in the Rac gain of function mutants, the cellular consequences are twofold: firstly, neurons fail to project their axons correctly and secondly neuronal migration is, at least in some cases, downregulated. The discovery that acetylation affects α-tubulin levels by regulating its turnover is important, since the dynamics of MT may also be altered in a system where α-tubulin is “hyperacetylated”. This may be the result of MTs being too short or prone to depolymerization. Whilst a regulation in number of MT cross-sections was not seen in the *mig-2(gf)* mutant, it was possible to suppress its phenotypes by either raising the stability of the MTs with taxol or by mimicking an α-tubulin accumulation by blocking the proteasome. This supports the hypothesis that the regulation of α-tubulin levels affects the dynamics of MTs. In contrast, if acetylation is reduced, as seen in the *elpc-1* mutants, then the length of MTs was probably increased. This evidence was supported by the observed resistance to the depolymerizing drug nocodazole and the increased sensitivity to taxol and proteasome inhibitor. In addition, a significant change in neuron shape was seen in *elongator* mutants. Altogether, the results advocate that the formation of stable MTs requires the fine-tuning of α-tubulin levels. Moreover, since Rac GTPases are upstream of these effects, it is possible that they contribute to the coordination of multiple events that lead to changes in cell shape, namely: a) actin cytoskeleton dynamics, b) stabilization of MT ends and c) fine-tuning of MT dynamics via regulation of α-tubulin turnover.

A further aspect is that axons of *mig-2(gf)* mutants do not only elongate less, but also show misguidance phenotypes [21]. Since acetylation of α-tubulin affects vesicle loading and transport, it is conceivable that extracellular axon guidance cues might be deregulated by transport problems due to incorrect acetylation of MTs [6]. Recently it has been shown that *mig-2* and *vab-8* (kinesin-like motor protein) are required to regulate the correct subcellular localization of UNC-40 (a homolog of the netrin receptor) which specifies cell polarity of neurons [51]. In addition, the authors showed that UNC-40 was mislocalized in a *mig-2* gain of function mutant. Of course MTs were hyperacetylated in that background, as shown by the results presented here. This leads to the assumption that UNC-40 was in fact mislocalized due to the improper loading and/or velocity of VAB-8 dependent vesicles. A more direct involvement of elongator complex in the regulation of intracellular trafficking that is critical for cell shape remodeling during migration and terminal branching of mouse cortical neurons (corticogenesis) has recently been shown [36]. In summary, this adds further evidence that acetylation may affect neuronal shapes by regulating a) the dynamics of MTs and their propensity to be stabilized via the turnover of α-tubulin and b) the loading and transport of vesicles (and their cargo).

### Stability and function of microtubules

The *elongator* mutants were shown to be resistant to the MT depolymerizing drug nocodazole. Based on this alone, one may conclude that MTs are more stable in *elongator* mutants, however further experiments showed that the resulting increase of α-tubulin levels also altered the dynamics of MTs. This can be confused with the notion that the “stability” of MTs requires an involvement of active stabilizing factors (such as +TIPs) which in the end change the half life of MTs [6],[50]. Although not the subject of this study, the possible link between *elongator* and +TIPs is an interesting point that should be investigated in the future.

Others have shown that changes in acetylation levels (mostly achieved by blocking the α-tubulin deacetylase HDAC6) modify the stability of MTs [8],[52]. Indeed, the experiments presented here confirm this point of view, and in addition allow a re-interpretation, namely that their findings are likely a result of the α-tubulin pool being regulated and not due to the direct regulation of MT stability.

### Protein turnover and acetylation

Deregulation of acetylation not only affects vesicle dynamics but also α-tubulin turnover (summarized in model Figure 8F). Indeed, this is important for cellular function as revealed by the movement defect of the *elongator*; *α-tubulin* double mutant. It is noteworthy that the defects can only be seen when crossing structural mutants of α-tubulin and not regulative or null mutants (Figure 3B–3D, Table S1). These defects may thus be the consequence of the accumulation of damaged α-tubulin, which in turn alters MT and neuronal function. In contrast, precise regulation of the levels of functional α-tubulin is required in the suppression experiments, since all mutations in α-tubulin abolish suppression. This provides further evidence that acetylation is important for the regulation of α-tubulin levels. Besides this work, a significant and growing amount of studies identifies acetylation of various proteins as a key regulator for their turnover. A very interesting finding is that molecules that participate in protein deacetylation, for example HDAC6, can directly interact with CDC48, a factor required for quality control and polyubiquitination of substrates. This reveals a link between control of acetylation states, active deacetylation and degradation [53],[54].

### Amyotrophic Lateral Sclerosis and Familial Dysautonomia

Recently it has been shown that elp3 was linked to neurodegenerative diseases and more specifically to Amyotrophic Lateral Sclerosis (ALS) in three families. ALS is the most common adult onset human motor neuron disease typically resulting in death from respiratory muscle weakness within three years [55]. Furthermore, mutations that cause tissue-specific exon skipping thereby truncating human Elp1 result in the autosomal recessive Familial Dysautonomia (FD), one of the most frequent hereditary neuropathies [39],[56]. Affected individuals are born with a reduced number of neurons within the autonomic and sensory nervous system. However, penetrance of the FD mutation is typically incomplete and a low level of full-length IKAP protein can prevail in brain tissues from FD patients [57],[58]. DNA microarray analysis on human cells revealed that the depletion of Elongator (via RNAi of ELP1) in fibroblasts modulates the expression of genes, several notably linked to motility and migration. This led to the conclusion that impaired cell movement within the nervous system might be involved in the neuropathology of FD patients [59]. Recently, a mouse knock out system showed that complete ablation of ELP1 leads to embryonic lethality due to defects in neurolation and blood vessel development [60]. As the worm knock-out of *elpc-1* is viable, it is arguably at present, the most suitable system to study and model the ELP1 function in neurons. Indeed, it may at first seem surprising that the defects in the worm nervous system are more similar to the human disease, while the mouse model has such a severe phenotype. However, several differences within the systems may offer plausible explanations. In humans, ELP1 is only downregulated, rather than completely deleted as in the mouse ELP1 experiment. Moreover, whilst only one α-tubulin is acetylable in the nematode, namely MEC-12/α-tubulin (expressed exclusively in neurons, which are not critical for survival as in mouse), all vertebrate α-tubulins possess the acetylatable lysine 40. Therefore regulation of vertebrate turnover of α-tubulin may have a greater impact at the cellular and organismal level and contribute to the severe phenotype observed in the knock out mouse. The mouse phenotypes may all be a result of defective cytoskeletal dynamics, such as reduced polarization and/or vesicular transport due to the downregulation of α-tubulin acetylation. The results presented here offer exquisite support for this line of argumentation, but clearly need further investigations into the transcriptional function of the *elongator* in vertebrates.

A further aspect is the degeneration of neurons present in FD patients as well as in ALS patients. In the worm model α-tubulin turnover is regulated through acetylation. Degenerative aspects may arise from deregulation of transcription as both ELP1 (involved in FD) and Elp3 (involved in ALS) are part of the same transcriptional *elongator* complex. Nevertheless, accumulation of “hypoacetylated” targets may be an additional explanation, since acetylation is important in the regulation of protein turnover. In addition, the reduced transport along MTs might lead to an accumulation of proteins destined for degradation. Indeed, the results show that transport is hampered in *elongator* mutants (Figure 4), that the *elongator* regulates α-tubulin acetylation and that downregulation of acetylation leads to accumulation of α-tubulin which in turn alters MT dynamics. Accumulation of α-tubulin, as well as the resulting changes in MT dynamics, may constitute an additional stress leading to degeneration and cell death.

### Crosstalk between microtubule and histone acetylation

The HAT activity of the *Elongator*, which is directed toward histone H3, has been proven in various systems to be crucial for transcription and affects genes important for cell movement in human fibroblasts [59]. Furthermore, it has been speculated that defects in this cellular function may underlie FD. In normal cells (fibroblasts and neurons) cell migration is always the concerted interaction between transcription and cytoskeletal dynamics. In this study α-tubulin was identified as an additional genetic target of the *elongator*. By acetylating histone H3 and regulating α-tubulin acetylation, it is conceivable that the *elongator* links and “synchronizes” the two functions to modulate the migration event. Rac GTPases may be involved in the response to outer signals and by doing so control the function of the *elongator*. To date, nothing is known about the regulation of *elongator* activation. This study highlights that acetylation in *mig-2(gf)* mutants is increased. However whether this is due to the activation of pathways leading to acetylation regulated by the *elongator*, to the downregulation of HDAC-6 function or an indirect consequence of upregulation of stabilizing +TIPs still awaits to be unraveled.

### Conclusion

This study uncovers that the *elongator* is linked to the regulation of α-tubulin acetylation. In addition the results presented here indicate that *elongator* is not only important for vesicle transport but also for α-tubulin turnover. This in turn seems to affect MT dynamics. These observations are instrumental for the introduction of a novel point of view, ultimately explaining how neuronal function is perturbed in neuropathies of Amyotrophic Lateral Sclerosis and Familial Dysautonomia. These new insights offer intriguing cues that targeting microtubules may lead to changes in the design of future therapies.

## Material and Methods

### *C. elegans* strains

Worms were handled according to standard procedures [61]. Strains: N2 (wt), *mig-2(mu28)X*, *mig-2(gm38)X*, *mig-2(gm103)X*, *rac-2(ok326)IV*, *ced-10(n1993)IV*, *mec-12(u76)III*, *mec-12(u63)III*, *mec-12(e1605)III*, *mec-12(e1607)III*, *unc-29(e1072)I*, *goa-1(n363)I*, *unc-64(e246)III*, *unc-104(e1265)II*, *mec-3(e1338)IV*. *elpc-1(ng10)I* and *elpc-3(ng15)V* were generated by ourselves: *elpc-1(ng10)I* by TMP/UV mutagenesis and *elpc-3(ng15)V* by tilling. *elpc-3(tm3120)V* was kindly provided by S. Mitani.

### Expression constructs and generation of transgenic strains

GABA-ergic motoneurons were visualized by crossing the strain *juIs76[Punc-25::gfp]*, analogously the CAN neuron was marked using *lqIs4[Pceh-10::gfp]* (gifts from E. A. Lundquist). Dense core vesicles were visualized using the strain BL5752 bearing the double insertion: *inIs181[ida-1::gfp]* and *inIs182[ida-1::gfp]*. Transgenics made by ourselves: (plasmid GU327): *ngEx2[elpc-1::gfp]* (50ng co-injected with 75ng of *ttx-3::gfp*), (plasmid GU329): *ngEx8[Ex[Punc-119::elpc-1::gfp]* (50ng co-injected with 50ng of *ttx-3::gfp*), (plasmid GU330): *ngEx43[Prab-3::elpc-1::gfp]* (20ng co-injected with 80ng of *ttx-3::rfp* and 20ng of *lin-15*), (plasmid GU446): *ngEx94[Prab-3::elpc-1-FD::gfp]* (20ng co-injected with 80ng of *ttx-3::rfp* and 20ng of *lin-15*), (plasmid GU320): *ngEx19[elpc-3::gfp]* (20ng co-injected with 80ng of *ttx-3::gfp*) and (plasmid GU448): *ngEx33[Prab-3::elpc-3]* (20ng co-injected with 80ng of *ttx-3::rfp*) (see Figure S3); (construct GU326): *ngIs14[hdac-6::gfp]I* (20ng co-injected with 80ng of *ttx-3::gfp*) (wormbase.org: “HDAC-6” = F41H10.6c), (construct GU312): *ngIs9[Pmec-12::gfp::mec-12]III* (5ng co-injected with 80ng of *ttx-3::rfp* and 20ng of *lin-15*), (construct GU331): *ngIs10[Pceh-10::gfp::mec-12]X* (20ng co-injected with 50ng of *ttx-3::rpf* and 50ng of *lin-15*) and (construct GU314): *ngIs11[Pmec-12::gfp::mec-12(K40Q)]IV* (5ng co-injected with 80ng of *ttx-3::rfp* and 20ng *lin-15*) (see Figure 6A–6C). The construct GU314 was also used for the differential expression of mec-12 in Figure 4A: *ngEx98* showed no detectable GFP signal (control strain for no expression of *mec-12::gfp*), *ngEx99* and *ngEx100* showed weak, *ngEx101* medium and *ngEx102* strong expression of *mec-12::gfp* (3ng co-injected with 80ng of *ttx-3::rfp* and 20ng *lin-15*). Constructs for nuclear or cytoplasmic expression of *elpc-3*: (construct GU449): *ngEx103* [Prab-3::NLS::elpc-3::gfp] (containing the NLS sequence from the Fire Lab vector kit) (20 ng co-injected with 80 ng of *ttx-3::rfp* and 20 ng of *lin-15*), (construct GU450): *ngEx104* [Prab-3::NES::elpc-3::gfp] (20 ng co-injected with 80 ng of *ttx-3::rfp* and 20 ng of *lin-15*). Constructs for ectopic expression of *mec-12*, *elpc-1* and *elpc-3* were designed using the promoter regions of *ceh-10* for CAN neuron expression [21], *unc-119* and *rab-3* for panneuronal expression (Figure S4). Plasmids GU327, GU329 and GU330 contain PCR amplified *elpc-1* cDNA cloned into pPD95.75 using the *Pst*I and *Xma*I sites. *Prab-3::elpc-1-FD::gfp* (GU446) was obtained deleting the 3′ end of *Prab-3::elpc-1::gfp* (GU330) using the convenient *Hpa*I site (in the cDNA) and the *Msc*I site (in the vector) and blunt ligated, in frame, with *gfp* (Figure S4). GU320 contains the whole *elpc-3* operon amplified by PCR and cloned into the *Xma*I site of pPD95.75. GU448 contains the *elpc-3* cDNA under the control of the *rab-3* promoter cloned into the *Not*I *Xma*I site of pPD95.75 vector. The NLS and NES versions of *elpc-3* were obtained by PCR amplification of the respective sequences and cloned into vector GU448. The coding sequence for the NES was: CTT CCA CCA CTC GAG AGG CTT ACG CTT. GU326 was obtained by PCR amplification of F41H10.6c (long isoform of HDAC-6) and cloning into pPD95.75 using the *Sph*I and *Xba*I sites. GU312 contains *mec-12* genomic locus (including the putative promoter) cloned into *Pst*I and *Kpn*I sites of pPD117.01, followed by insertion of *gfp* into *Bgl*II site in-frame with *mec-12*. *Pmec-12::gfp::mec-12(K40Q)* (GU314) was obtained by standard point mutation method using *Pmec-12::gfp::mec-12* as template and subsequently sequenced. GU331 contains *ceh-10* promoter inserted via *Sal*I and *Bam*HI sites into pPD117.01 and subsequently *mec-12* cDNA was added with *Xba*I site into the compatible *Nhe*I site. Primer sequences used for PCR amplifications are available upon request. All expression constructs were cloned into the vector pPD95.75 except for *mec-12* and *mec-12(K40Q)* cloned in pPD117.01 (kind gift from A. Fire).

### Worm mutagenesis and integration of constructs

The *elpc-1(ng10)* deletion allele was performed as described [62]. *elpc-3(ng15)* was isolated by tilling as described [63]. Integration of constructs GU326, GU312, GU331 and GU314 was obtained by UV-irradiation (30,000J) of L4 stage worms. Fertile young adults were singled the next day and subsequently starved for 2 weeks to lose the non-integrated extrachromosomal array. Starved worms were transferred onto fresh growth plates and scored for stable inheritance of the respective *ttx-3::rfp* marker. Integrants were outcrossed twice then mapped by crossing with strain DA438 containing markers for each chromosome (kindly provided by CGC and constructed by L. Avery).

### Protein biochemistry

Worms grown in liquid at 20°C to the appropriate stage were collected in buffer A (20 mM Tris pH 7.4, 200 mM NaCl, 10% (v/v) glycerol, 1% Triton) and stored at −80°C. Protein extracts were prepared adding 1 mM PMSF, 1× complete protease inhibitor (Roche) and 300 nM TSA (Sigma T8552). Acid-washed glass beads (Sigma G8772) and FastPrep-24 sample preparation system (MP Biomedicals) were used to homogenize worms (4.0 m/s for 45 seconds at RT). After centrifugation at 4°C, the protein concentration of the supernatants was determined by Bradford assay (Biorad) and 15 µg total protein were resuspended in Laemmli buffer. Proteins separated on 10% SDS-PAGE gels were detected by immunoblotting using ECL. Worm extracts for immunoprecipitation were prepared in HNNG buffer (15 mM Hepes pH 7.5, 250 mM NaCl, 1% NP- 40, 5% glycerol, 1 mM PMSF, 10 mM sodium butyrate, -Sigma B5887-, 300 mM TSA and 1× protease inhibitors cocktail). Lysates (1–2 mg/ml) were incubated overnight with 1 µg of antibody at 4°C. Immunocomplexes were collected with protein A plus sepharose beads (Amersham Biosc), protein G plus sepharose beads (Zymed), anti-α-tubulin- or anti-ELPC-1-conjugated agarose beads, sequentially washed at 4°C with HNNG buffer and finally resuspended in Laemmli buffer. anti-α-tubulin and anti-ELPC-1 crosslinked resins have been produced by Cogentech (Consortium for Genomic Technologies, Milan, Italy) using standard procedures. Antibodies used for western blots and/or immunoprecipitations: anti-α tubulin (Sigma T5168), anti-acetylated-α-tubulin (Sigma, T6793), anti-actin (MP biomedicals, #69100), anti-GFP (Torrey Pines Biolabs Inc, TP401) and anti-Ubi (Upstate, # 05-944). Rabbit polyclonal anti-ELPC-1 antibody was produced by Cogentech. anti-rabbit or anti-mouse IgG HRP-conjugated antibodies were both from Cell Signaling Tech (# 7074 and # 7076, respectively). Western blots were performed at least twice to confirm the results.

### TEM, GFP quantification and immunolocalization, Dense Core Vesicles velocity

TEM was performed using standard procedures. Briefly, worms were washed in M9 and anesthetized in 8% ethanol in M9 for 5 min. They were placed in a fixative (2.5% glutaraldehyde, 1% paraformaldehyde in 0.1M sucrose, and 10 mM PBS, pH 7.4), cut open with a needle at the anterior and posterior ends, and fixed for 2 h. Worms were embedded in 2% agarose, cut into small blocks, and washed three times in PBS. Subsequently, pieces were fixed with a second solution (1% osmium tetroxide, 1.5% potassium ferrocyanide in PBS) for 2 h and washed three times in water. Worms were stained with 1% uranyl acetate for 1 h. Samples were dehydrated in ethanol (10 min in 50% ethanol, 10 min in 70% ethanol, 10 min in 90% ethanol, and 10 min in 100% ethanol) and acetone (10 min). Blocks with worms were embedded in Epon resin (Fluka, Buchs, Switzerland): first in Epon-acetone (1∶1) for 1–2 h and then in pure resin for 2–4 h. Samples polymerized for 24–48 h at 60°C and in 60-nm sections were prepared with Ultracut E. Sections were stained in uranyl acetate for 60 min and then 2 min in Millonig's lead acetate stain. Pictures were taken on Philips Morgagni 80 KV microscope (Eindhoven, The Netherlands) [64].

GFP signal was quantified by taking pictures of ALM or CAN neuronal body of anesthetized L1 at 63× magnification at fixed exposure time (2 sec.) The total signals were measured using ImageJ software. Whole mount staining of acetylated MEC-12/α-tubulin using the specific monoclonal antibody (see below) was performed according to previous protocols [10]. Cy3 conjugated secondary antibodies from Jackson Lab. were used. Primary antibody dilution: 1/500. Secondary antibody dilution: 1/200. Analysis of dense core vesicles was performed as described [43]. Immunolocalizations were repeated 3× per genotype.

### Interpretation of TEM data

In Figure 7C we show the two sets of measurements for the number of intersections of microtubules on eight independent sections of ALM in a wild type and an *elpc-1(lf)* mutant. In wt the average number of intersections and its standard deviation are μ_1_ = 67 and σ_1_ = 9; for *elpc-1(lf)* μ_2_ = 85 and σ_2_ = 5. The means are statistically different: μ_1_ differs from μ_2_ (one-sided t test, P = 2·10^−4^; 11–Inf is 95% confidence interval for μ_2_−μ_1_). These measurements confirm that the two individuals of different genotype show similar MT intersection levels than the respective ones in Figure 7B. Based on these data, homogeneity of variances cannot be discarded (one-sided F test, P = 0.1). Interpretation model: Assuming that microtubules have a fixed length h, parallel to the axis of the axon and distributed uniformly along an axon of a given length L leads to following calculations: the probability for a microtubule to cross any section is p = h/L and follows the Poisson distribution (h/L equals the ways a “stick” of length h can be placed in a longer “tube” of length L). Performing N trials of a Poisson process with probability p, the expected value and the variance of the number of successes for a random variable X are E(X) = Np and VAR(X) = Np(1−p), respectively. Thus, if we have m microtubules, the expected value for the number of intersections is E(X) = mh/L, while the variance is VAR(X) = mh/L(1−h/L). Since the *elpc-1(lf)* mutant has, on average, more microtubules intersections than wild type, using E(X) = mh/L we obtain the condition m_2_h_2_>m_1_h_1_ (1 = wt; 2 = *elpc-1(lf)*). In other words, *elpc-1(lf)* have more microtubules, or the microtubules are longer, or both. It seems unlikely that the main change is the number of microtubules, because the assumption that microtubule length is unchanged, i.e. h_2_ = h_1_ and m_2_>m_1_, requires VAR_2_(X)>VAR_1_(X), i.e. σ^2^_2_>σ^2^_1_. The later is unsupported by the measurements.

### Movement tests and drug treatments

Body bends per minute: Worms grown at 20°C to L4/young adult stage were placed into water. Body bends were counted for 1 minute. Blind scoring was not applied to these tests, because they were highly reproducible and not subjective. The number of body bends per minute for wt worms in our tests reproduced published levels [65]. Drug tests: Standard NGM plates were supplemented with 0.1–0.5 µM nocodazole, 1–5 µM taxol [46], 0–75 µM leptomycin B [42] or 0–40 µM lactacystin [48]. DMSO [for nocodazole and taxol] or water [for leptomycin and lactacystin] alone were used as a control. Worms were grown in the presence of the respective drug and body bend assays were carried out on the F1. The Aldicarb tests were performed as described [44]. Concentration used: 0–40 µM. Modifications to the original protocol: worms were exposed only 1h (instead of 4h) and the test was performed in M9 buffer (this modified procedure increased the sensitivity). Levamisole test was performed as described [44]. Aldicarb and levamisole tests were repeated 5 times using each time 12 animals calculating the average and error. The sensitivity of wt worms when exposed 4h to aldicarb and in the levamisole test reproduced the published data (not shown, [44]). Touch sensitivity: young adult worms were touched 10 times with an eyelash, alternating between the anterior and posterior part of the body. Between touches worms were given 1 min to recover. Positive response by movement away form the stimulation was scored for 10 independent worms per strain.

### Apoptotic bodies and distal tip cell migration phenotype analysis

Performed as described [21],[40].

### ACh measurement

Young adult worms were flash frozen in water (1000 worms per pellet). Extraction was performed in 0.2 M perchloric acid (PCA) solution with 100 mM EDTA. Ethylhomocholine (0.1 µM) served as internal standard. To homogenize the worms the FastPrep®-24 sample preparation system with beads was used as described above. After centrifugation, the pH of the supernatant was neutralized using 1M KHCO_3_ and purified using 0.45 mm pore size filters. Final extracts were stored at −80°C and analyzed on an electrochemical detector HTEC-500 (Eicom Co. Kyoto, Japan) as described [66].

### Statistical analysis

If not otherwise stated, statistical significance was performed by two-tailed unpaired Student's t-test. P value>0,05 was scored as “ns” (***n***ot ***s***ignificant).

## Supporting Information

Figure S1 *C. elegans* α-tubulin alignments, *mec-12* expression, movement and acetylation defects are not dependent on touch neurons only. (A) Alignment of *C. elegans* α-tubulins identifying MEC-12 as the only one having the epitope with lysine 40. This epitope, when acetylated on Lys 40 is recognized by a specific monoclonal antibody and is common to all the α-tubulins in vertebrates. Outside the shown area, all α-tubulins of *C. elegans* are nearly identical to each other and to the vertebrate homologue (not shown) (B) Translational construct used to determine *mec-12* expression. We see expression in almost all neurons (C–G), which is confirmed by literature (using transcriptional constructs) [10] [Fukushige et al.]. (C) Fluorescence micrograph: overview in L4 larva of GFP::MEC-12 expression. Touch neurons are depicted. Bar: 20 micrometers. (D) Expression of GFP::MEC-12 in tail neurons of an adult animal. Touch neuron is depicted. Bar: 10 micrometers. (E) Expression of GFP::MEC-12 in head neurons of a young adult. Lines indicate position of the nerve ring. (F) Equivalent to (E) using DIC. Bar: 20 micrometers. (G) Ventral nerve cord of a young adult. Arrowheads: motoneurons expressing GFP::MEC-12. Bar: 10 micrometers. (H) Scheme of the position of the 6 touch neurons, explaining that they are not specified in a *mec-3(e1338)* mutant [38]. (I) Movement by body bends per minute of adult animals of different genotypes in wt or *mec-3(e1338)* background showing no difference in movement phenotypes. (J) Western blot analysis of MEC-12/α-tubulin acetylation. Developmental stages of worms are indicated. Total α-tubulin served as loading control. Panels show wt (N2) and *mec-3(e1338)* backgrounds as indicated. Comparative western blots were carried out and representative blots for the two strains are shown. Acetylation is present in *mec-3(e1338)* mutants although touch neurons are not specified, therefore other neurons than touch neurons express acetylated MEC-12/α-tubulin. P values: ***<0,001. Error bars: SEM. (Fukushige T, Siddiqui ZK, Chou M, Culotti JG, Gogonea CB, et al. (1999) MEC-12, an alpha-tubulin required for touch sensitivity in *C. elegans*. J Cell Sci 112 ( Pt 3): 395–403.)
(2.38 MB TIF)

Figure S2 Homology of *elongator* proteins. (A) Table summarizing the degree of homology between human and worm elongator proteins. (B) Alignment of *C. elegans*, yeast and human elp3 proteins to denote the high degree of identity and showing the position of the *elpc-3(ng15)* R→C point mutation.
(1.58 MB TIF)

Figure S3 Summary of constructs and mutations of *elpc-1* and *elpc-3*. *unc-119* and *rab-3* promoters were chosen for neuronal expression [Maduro et al., Nonet et al.]. *elpc-1*: genomic structure of the gene with predicted domains, truncation in FD patients, structure of the deletion ng10, RNAi probe and constructs. *elpc-3*: genomic structure of the gene with predicted domains, structure and location of the mutants, the point mutation was produced to confirm that the deletion allele *tm3120* has no effect on overall expression of the operon 5324 (in green). Both mutants can be rescued by *elpc-3* cDNA constructs (see text). RNAi probe and constructs. (Maduro M, Pilgrim D (1995) Identification and cloning of unc-119, a gene expressed in the Caenorhabditis elegans nervous system. Genetics 141: 977–988.) (Nonet ML, Staunton JE, Kilgard MP, Fergestad T, Hartwieg E, et al. (1997) Caenorhabditis elegans rab-3 mutant synapses exhibit impaired function and are partially depleted of vesicles. J Neurosci 17: 8061–8073.)
(0.65 MB TIF)

Figure S4 Response to light body touch of different mutants and MTs in an axon model. (A) *elpc-1* and *elpc-3* have no touch response phenotype. *mec-12(u76)* are touch insensitive (see also [10]. (B) Model explaining how longer MTs can give a higher average in MT count per cross-section. P values: ***<0,001. Error bars: SEM.
(0.24 MB TIF)

Figure S5 Details of immunohistochemical detection of acetylated *mec-12*/α-tubulin staining and scheme of the whole genome RNAi screen to find suppressors of *mig-2(gf)*. (A) [left] Details on acetylated α-tubulin signals in N2(wt) and *mig-2(gm38)*. Lines (1–4) where the profile was measured to quantify the signal. Bar = 10 micrometers. Upper arrow: nerve ring, lower arrow: sensilla. Magnified detail of head-staining in *mig-2(gm38)*. [right] Quantification of immunofluorescence. (B) Quantification of the western blot in Figure 2B. The relative signals were adjusted using the tubulin loading control.
(0.91 MB TIF)

Table S1 Description of known molecular basis of different *mec-12* alleles used in this study.
(0.03 MB DOC)

Table S2 Formation of apoptotic bodies in the gonad is normal in elongator mutants. The number of gonadal arms that were counted is indicated (n*); ns = not significant.
(0.07 MB DOC)

Table S3 Distal tip cell migration phenotype in *elongator* mutants. Yellow = phenotypes that are indistinguishable from *mig-2(mu28)*; pink = phenotypes that are indistinguishable from *mig-2(mu38)*; white = wild type.
(0.06 MB DOC)
