# Supplementary figures and images for: The Caenorhabditis elegans Elongator Complex Regulates Neuronal α-tubulin Acetylation
Source: PLoS Genet. 2010 Jan 22;6(1):e1000820. doi: 10.1371/journal.pgen.1000820 (PMC2809763; doi:10.1371/journal.pgen.1000820)

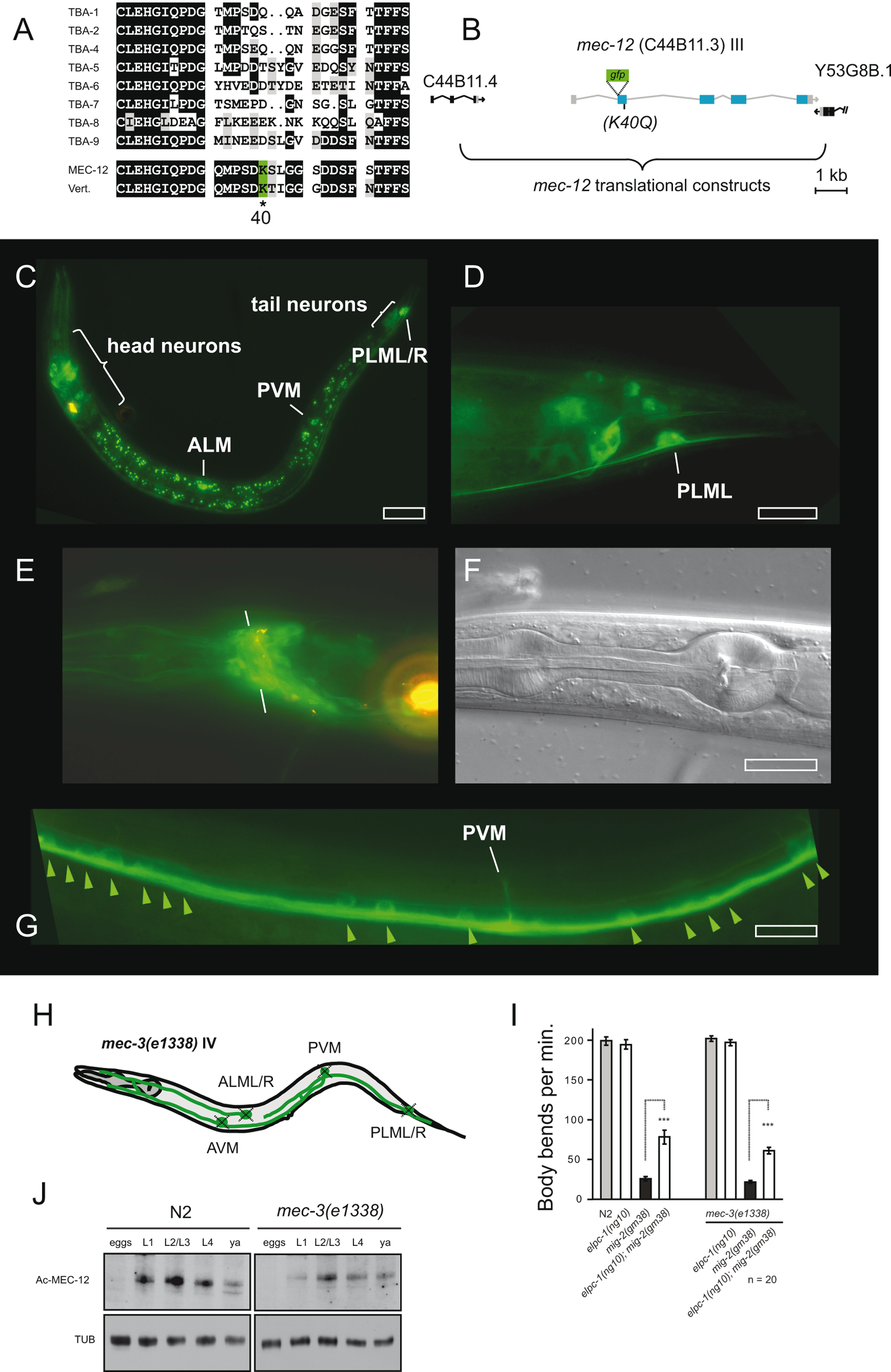

Supplement: Figure S1 — C. elegans α-tubulin alignments, mec-12 expression, movement and acetylation defects are not dependent on touch neurons only. (A) Alignment of C. elegans α-tubulins identifying MEC-12 as the only one having the epitope with lysine 40. This epitope, when acetylated on Lys 40 is recognized by a specific monoclonal antibody and is common to all the α-tubulins in vertebrates. Outside the shown area, all α-tubulins of C. elegans are nearly identical to each other and to the vertebrate homologue (not shown) (B) Translational construct used to determine mec-12 expression. We see expression in almost all neurons (C–G), which is confirmed by literature (using transcriptional constructs) [10] [Fukushige et al.]. (C) Fluorescence micrograph: overview in L4 larva of GFP::MEC-12 expression. Touch neurons are depicted. Bar: 20 micrometers. (D) Expression of GFP::MEC-12 in tail neurons of an adult animal. Touch neuron is depicted. Bar: 10 micrometers. (E) Expression of GFP::MEC-12 in head neurons of a young adult. Lines indicate position of the nerve ring. (F) Equivalent to (E) using DIC. Bar: 20 micrometers. (G) Ventral nerve cord of a young adult. Arrowheads: motoneurons expressing GFP::MEC-12. Bar: 10 micrometers. (H) Scheme of the position of the 6 touch neurons, explaining that they are not specified in a mec-3(e1338) mutant [38]. (I) Movement by body bends per minute of adult animals of different genotypes in wt or mec-3(e1338) background showing no difference in movement phenotypes. (J) Western blot analysis of MEC-12/α-tubulin acetylation. Developmental stages of worms are indicated. Total α-tubulin served as loading control. Panels show wt (N2) and mec-3(e1338) backgrounds as indicated. Comparative western blots were carried out and representative blots for the two strains are shown. Acetylation is present in mec-3(e1338) mutants although touch neurons are not specified, therefore other neurons than touch neurons express acetylated MEC-12/α-tubulin. P values: ***<0,001. E [file pgen.1000820.s001.tif]

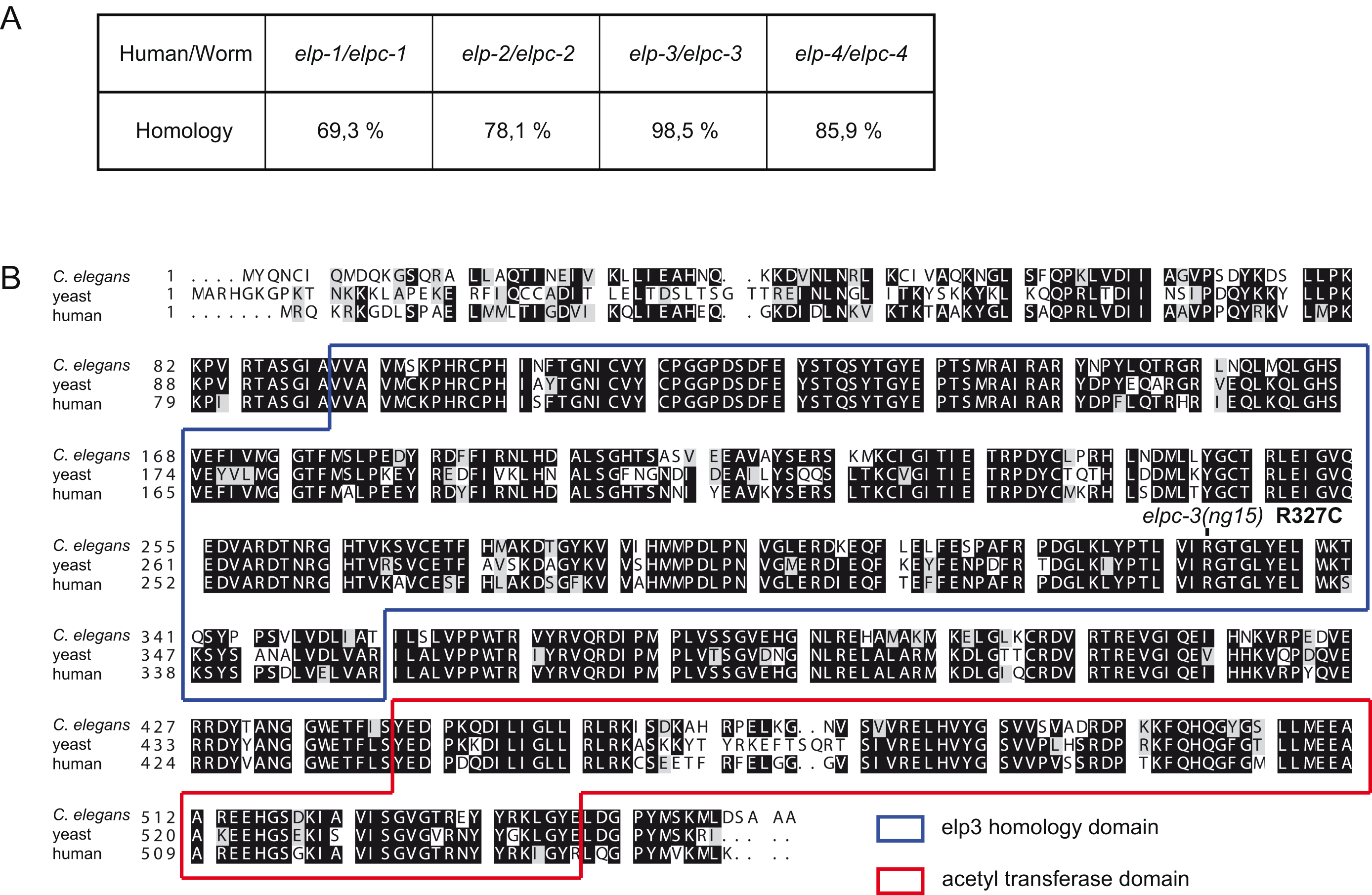

Supplement: Figure S2 — Homology of elongator proteins. (A) Table summarizing the degree of homology between human and worm elongator proteins. (B) Alignment of C. elegans, yeast and human elp3 proteins to denote the high degree of identity and showing the position of the elpc-3(ng15) R→C point mutation. (1.58 MB TIF) [file pgen.1000820.s002.tif]

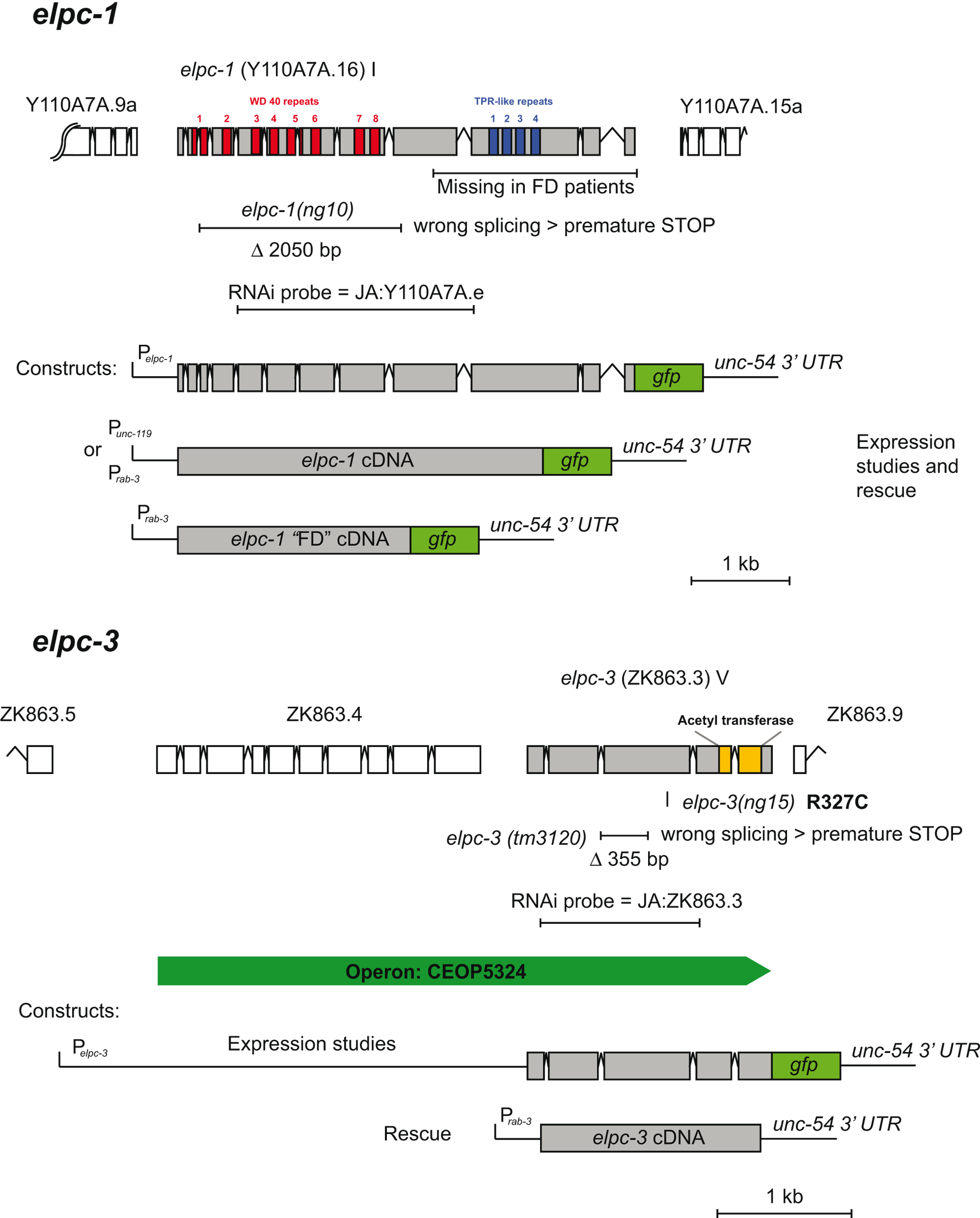

Supplement: Figure S3 — Summary of constructs and mutations of elpc-1 and elpc-3. unc-119 and rab-3 promoters were chosen for neuronal expression [Maduro et al., Nonet et al.]. elpc-1: genomic structure of the gene with predicted domains, truncation in FD patients, structure of the deletion ng10, RNAi probe and constructs. elpc-3: genomic structure of the gene with predicted domains, structure and location of the mutants, the point mutation was produced to confirm that the deletion allele tm3120 has no effect on overall expression of the operon 5324 (in green). Both mutants can be rescued by elpc-3 cDNA constructs (see text). RNAi probe and constructs. (Maduro M, Pilgrim D (1995) Identification and cloning of unc-119, a gene expressed in the Caenorhabditis elegans nervous system. Genetics 141: 977–988.) (Nonet ML, Staunton JE, Kilgard MP, Fergestad T, Hartwieg E, et al. (1997) Caenorhabditis elegans rab-3 mutant synapses exhibit impaired function and are partially depleted of vesicles. J Neurosci 17: 8061–8073.) (0.65 MB TIF) [file pgen.1000820.s003.tif]

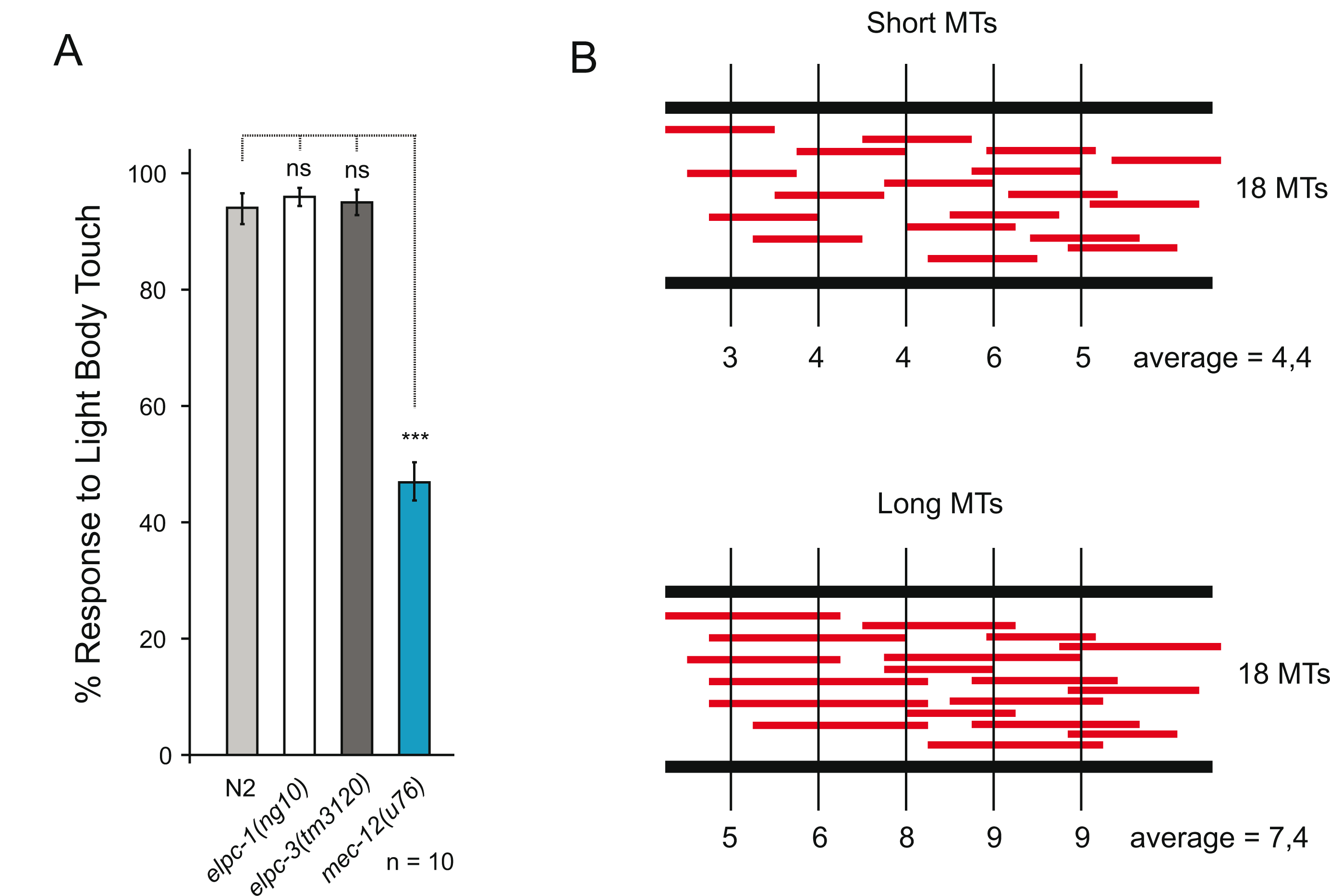

Supplement: Figure S4 — Response to light body touch of different mutants and MTs in an axon model. (A) elpc-1 and elpc-3 have no touch response phenotype. mec-12(u76) are touch insensitive (see also [10]. (B) Model explaining how longer MTs can give a higher average in MT count per cross-section. P values: ***<0,001. Error bars: SEM. (0.24 MB TIF) [file pgen.1000820.s004.tif]

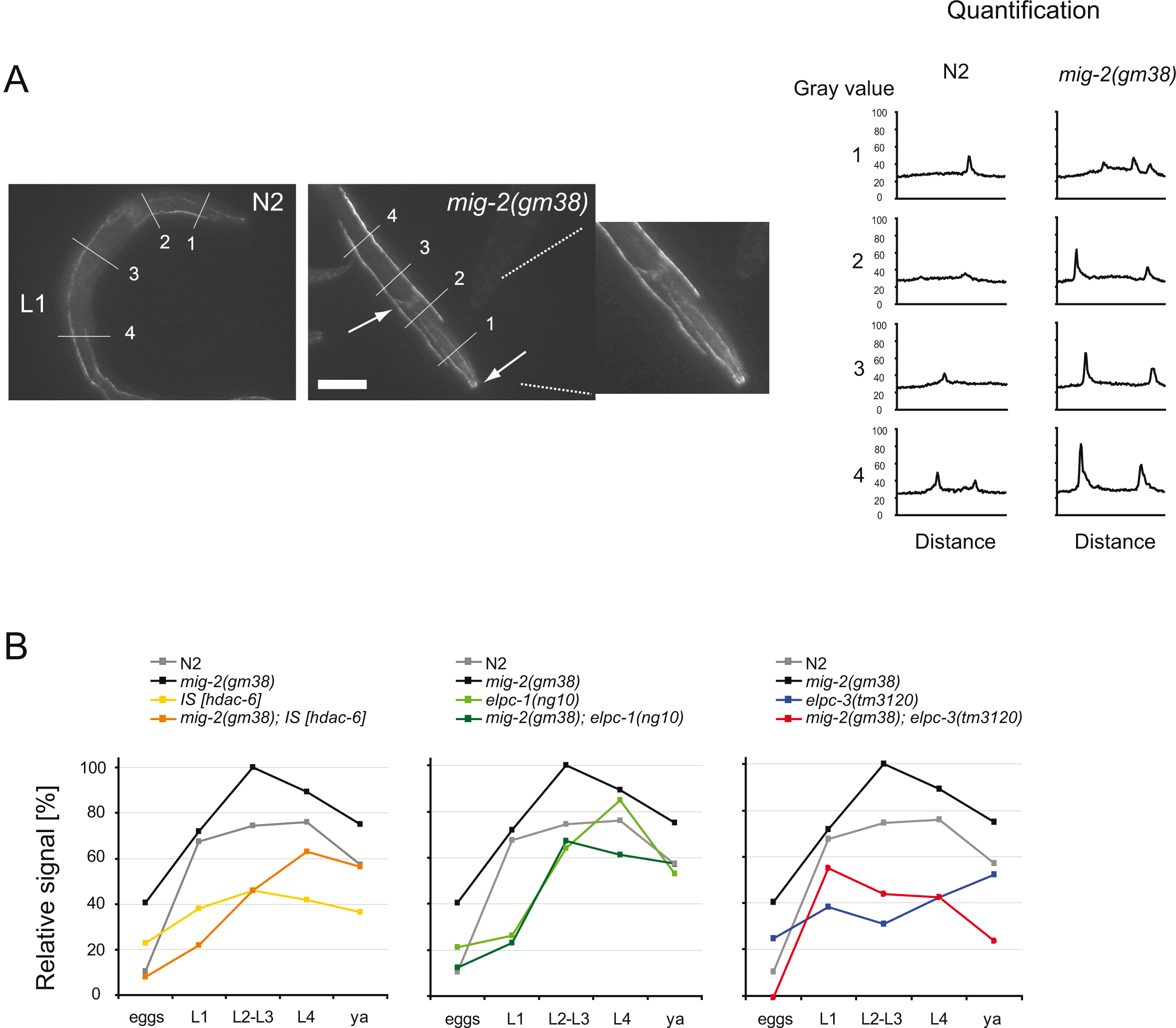

Supplement: Figure S5 — Details of immunohistochemical detection of acetylated mec-12/α-tubulin staining and scheme of the whole genome RNAi screen to find suppressors of mig-2(gf). (A) [left] Details on acetylated α-tubulin signals in N2(wt) and mig-2(gm38). Lines (1–4) where the profile was measured to quantify the signal. Bar = 10 micrometers. Upper arrow: nerve ring, lower arrow: sensilla. Magnified detail of head-staining in mig-2(gm38). [right] Quantification of immunofluorescence. (B) Quantification of the western blot in Figure 2B. The relative signals were adjusted using the tubulin loading control. (0.91 MB TIF) [file pgen.1000820.s005.tif]
