# Supplementary material for: The Caenorhabditis elegans Elongator Complex Regulates Neuronal α-tubulin Acetylation
Source: PLoS Genet. 2010 Jan 22;6(1):e1000820. doi: 10.1371/journal.pgen.1000820 (PMC2809763; doi:10.1371/journal.pgen.1000820)
Supplement: Table S1 — Description of known molecular basis of different mec-12 alleles used in this study. (0.03 MB DOC) [file pgen.1000820.s006.doc]

**Table S1**

**Description of *mec-12* alleles**

| MEC-12 allele: | Mutation: | Description: | Sequence analysis: | Reference: |
| --- | --- | --- | --- | --- |
| *u63* | E415K | putative structural damage | [10] | [10] |
| *e1605* | no mutation found in the cDNA | unknown molecular damage; strong *mec* phenotype, probably regulative | [10], this study | [10], this study |
| *u76* | D69N | putative structural damage; near acetylation site | [10] | [10] |
| *e1607* | G144S | conserved residue necessary for GTP binding and dimerization with beta-tubulin, constitutes a putative null allele | this study | [Supporting Reference] |

Supporting Reference:

Zabala JC, Fontalba A, Avila J (1996) Tubulin folding is altered by mutations in a putative GTP binding motif. J Cell Sci 109 ( Pt 6): 1471-1478.
