# Supplementary material for: The Caenorhabditis elegans Elongator Complex Regulates Neuronal α-tubulin Acetylation
Source: PLoS Genet. 2010 Jan 22;6(1):e1000820. doi: 10.1371/journal.pgen.1000820 (PMC2809763; doi:10.1371/journal.pgen.1000820)
Supplement: Table S2 — Formation of apoptotic bodies in the gonad is normal in elongator mutants. The number of gonadal arms that were counted is indicated (n*); ns = not significant. (0.07 MB DOC) [file pgen.1000820.s007.doc]

**Table S2**

**Apoptotic Bodies**

| Genotype | Apoptotic Bodies per gonadal arm ± st. dev. | p value vs N2 | p value vs  *mig-2(mu28)* | p value vs  *mig-2(mu38)* | n* |
| --- | --- | --- | --- | --- | --- |
|  |  |  |  |  |  |
| N2 | 0,4 ± 0,1 |  |  |  | 25 |
|  |  |  |  |  |  |
| *mig-2(mu28)* | 0,3 ± 0,1 | ns |  |  | 25 |
| *mig-2(gm38)* | 0,6 ± 0,2 | ns |  |  | 25 |
|  |  |  |  |  |  |
| *elpc-1(ng10)* | 0,6 ± 0,1 | ns |  |  | 25 |
| *elpc-3(ng15)* | 0,5 ± 0,1 | ns |  |  | 25 |
| *elpc-1(ng10); elpc-3(ng15)* | 0,6 ± 0,2 | ns |  |  | 25 |
|  |  |  |  |  |  |
| *elpc-1(ng10); mig-2(mu28)* | 0,6 ± 0,1 | ns | ns |  | 25 |
|  |  |  |  |  |  |
| *elpc-1(ng10); mig-2(gm38)* | 0,7 ± 0,1 | ns |  | ns | 25 |
| *elpc-3(ng15); mig-2(gm38)* | 0,3 ± 0,1 | ns |  | ns | 25 |
| *elpc-1(ng10); elpc-3(ng15); mig-2(gm38)* | 0,4 ± 0,1 | ns |  | ns | 25 |
|  |  |  |  |  |  |
| *mec-12(u76)* | 0,5 ± 0,2 | ns |  |  | 25 |
| *elpc-1(ng10); elpc-3(ng15); mec-12(u76)* | 0,4 ± 0,1 | ns |  |  | 25 |
|  |  |  |  |  |  |
| *rac-2(ok326)* | 0,5 ± 0,1 | ns |  |  | 25 |
| *ced-10(n1993)* | 2,2 ± 0,4 | 0,0002 |  |  | 25 |
|  |  |  |  |  |  |
