# Supplementary material for: The Caenorhabditis elegans Elongator Complex Regulates Neuronal α-tubulin Acetylation
Source: PLoS Genet. 2010 Jan 22;6(1):e1000820. doi: 10.1371/journal.pgen.1000820 (PMC2809763; doi:10.1371/journal.pgen.1000820)
Supplement: Table S3 — Distal tip cell migration phenotype in elongator mutants. Yellow = phenotypes that are indistinguishable from mig-2(mu28); pink = phenotypes that are indistinguishable from mig-2(mu38); white = wild type. (0.06 MB DOC) [file pgen.1000820.s008.doc]

**Table S3**

**Distal Tip Cell Migration Phenotype**

| Genotype | % of gonad shape defects classified  as in Lundquist et al 2001 | | | n |
| --- | --- | --- | --- | --- |
|  | normal | no outgrowth | misguided |  |
|  |  |  |  |  |
| N2 | 100 |  |  | 50 |
|  |  |  |  |  |
| *mig-2(mu28)* | 81,4 |  | 18,6 | 102 |
| *mig-2(gm38)* | 95,1 | 1 | 3,9 | 102 |
|  |  |  |  |  |
| *elpc-1(ng10)* | 100 |  |  | 105 |
| *elpc-3(ng15)* | 100 |  |  | 100 |
| *elpc-1(ng10); elpc-3(ng15)* | 100 |  |  | 100 |
|  |  |  |  |  |
| *elpc-1(ng10); mig-2(mu28)* | 81,1 |  | 18,9 | 106 |
|  |  |  |  |  |
| *elpc-1(ng10); mig-2(gm38)* | 94,2 | 1 | 4,8 | 104 |
| *elpc-3(ng15); mig-2(gm38)* | 96,2 |  | 3,8 | 104 |
| *elpc-1(ng10); elpc-3(ng15); mig-2(gm38)* | 95,2 |  | 4,8 | 105 |
|  |  |  |  |  |
| *mec-12(u76)* | 100 |  |  | 100 |
| *elpc-1(ng10); elpc-3(ng15); mec-12(u76)* | 100 |  |  | 100 |
|  |  |  |  |  |
| *rac-2(ok326)* | 100 |  |  | 100 |
| *ced-10(n1993)* | 84,5 |  | 15,5 | 103 |
|  |  |  |  |  |
